# Prediction and quality evaluation of quality markers of *Gentiana scabra* Bunge. in treatment of liver injury

**DOI:** 10.3389/fphar.2025.1679981

**Published:** 2025-11-06

**Authors:** Jiayi Liu, Xuelin Yu, Jiayi Luo, Dan Wang, Haibo Yin

**Affiliations:** Liaoning University of Traditional Chinese Medicine, Dalian, China

**Keywords:** *Gentiana scabra* Bunge., liver injury, multivariate statistical analysis, quality markers, quality evaluation

## Abstract

**Introduction:**

*Gentiana scabra* Bunge. (GSB) has been demonstrated to exert nourishing effectson the liver and gallbladder. Notably, GSB extract has a marked therapeutic effect on liver injury (LI). However, the quality markers (Q-markers) of GSB associated with this effect remain unclear. Moreover, no study to date has evaluated the quality of GSB from different producing areas based on such Q-markers.

**Methods:**

The aim of this study was to identify the Q-markers of GSB that define its therapeutic efficacy against liver injury. This was achieved through establishing HPLC fingerprints and conducting “small molecule–protein” interaction screening, followed by verification using *in vitro* cell experiments. Finally, the identified Q-markers were quantified to assess the quality of GSB samples from different producing areas.

**Results:**

First, the HPLC fingerprints of 44 batches of GSB from differentproducing areas were established, revealing 25 common peaks. Principal component analysis (PCA) and Orthogonal Partial Least Squares Discriminant Analysis (OPLS-DA) of the 25 peak areas resulted in the identification of five components based on Variable Importance in Projection (VIP) values >1. Subsequently, these small-molecule components of GSB were screened for interaction with liver proteins, leading to the identification of three components with strong efficacy, namely, swertiamarin, gentiopicroside, and sweroside, which were designated as Q-markers of GSB associated with liver injury treatment. Furthermore, the therapeutic efficacy of these Q-markers was validated using a hydrogen peroxide-induced NCTC 1469 cell injury model. Finally, the Q-markers were quantified, and the quality of GSB samples from different origins was evaluated through PCA, entropy-weighted Technique for Order Preference by Similarity to Ideal Solution (TOPSIS), and Hierarchical Cluster Analysis (HCA) based on the quantification results.

**Discussion:**

The potential Q-markers of GSB were predicted through the integration of HPLC fingerprinting, pattern recognition, and small molecule–protein interaction analysis. Cell experiment results showed that swertiamarin, gentiopicroside, and sweroside significantly alleviated liver cell injury. Quantitative analysis of the Q-markers identified significant differences in their contents across GSB samples from different producing areas. This methodology provides a novel and comprehensive framework for defining Q-markers and evaluating GSB quality.

## 1 Introduction

Gentianae Radix et Rhizoma is derived from the dried roots and rhizomes of *Gentiana scabra* Bunge. (GSB), *Gentiana manshurica* Kitag., *Gentiana triflora* Pall., and *Gentiana rigescens* Franch. The first three are collectively termed “Guan Gentiana,” and the last one is known as “Jian Gentiana.” GSB is characterized by a bitter taste and a cold nature. It acts on the Liver Meridian (of Foot-Jueyin) and the Gallbladder Meridian (of Foot-Shaoyang), and has the effect of clearing heat, drying dampness, and purging fire from the liver and gallbladder. It is especially suitable for symptoms such as damp-heat jaundice, dull pain, and Yin itch (Committee National Pharmacopoeia, 2020). GSB has a long history of use in clinical practice. However, the increasing demand and overexploitation of wild GSB resources over recent years have resulted in quality variations. This uneven GSB quality stems from differences in producing areas, provenance, ecological environments, and cultivation methods. Traditional Chinese Medicine (TCM) is characterized by its multi-component and multi-target properties, and its quality is fundamental for ensuring stable efficacy and safe application. Consequently, establishing effective quality control for GSB, thus ensuring its consistency and stability, and associating quality control components with efficacy have become pressing needs. To comprehensively address TCM quality, Changxiao Liu proposed the concept of quality markers (Q-markers), which aims to effectively manage TCM safety and measurability. Q-markers are defined as inherent substances closely related to the functional properties of a TCM, possessing clear chemical structures that can be qualified and quantified (Liu et al., 2016). Currently, the Chinese Pharmacopeia specifies only a single-component, gentiopicroside, for addressing the evaluation of GSB quality. However, the clinical efficacy of TCM mainly depends on the characteristics of its multiple chemical constituents (groups), which underpins the research idea of “efficacy-based” TCM quality control (Chang et al., 2022). Consequently, the establishment of Q-markers is gradually evolving from the identification of single or a few compounds to the characterization of multiple active ingredients (Zhang et al., 2022).

Oxidative stress (OS) refers to an imbalance between the production of reactive oxygen species (ROS) and their clearance by the antioxidant system following endogenous or exogenous stimuli (Peluso et al., 2012). This disrupts cellular oxidation reactions and leads to molecular-level cellular damage. Studies have shown that OS can elicit changes in cell signaling and the cell cycle, impair cell transportation mechanisms, disrupt biological activity, and induce immune activation and inflammatory responses (Newsholme et al., 2016).

The liver is considered both an immune organ and the main detoxification organ in the body (Bogdanos et al., 2013). In a healthy state, liver cells maintain a dynamic balance. However, under oxidative stress or hypoxia, the liver becomes a primary target for free radical attack, leading to tissue abnormalities, liver cell damage, and, eventually, necrosis or apoptosis. At present, the pharmacologically active components of GSB responsible for its therapeutic effects against liver injury remain unclear, which limits its quality control and further development. Therefore, exploring how GSB mitigates the deleterious effects of oxidative stress on liver cells holds far-reaching significance for identifying the Q-markers of GSB in the treatment of LI.

Based on the concept of Q-markers and the principle of component quantifiability and effectiveness, we used high-performance liquid chromatography to establish fingerprints for GSB samples of different origins and detect their chemical components. The pharmacologically active components associated with the treatment of liver injury were further screened using fluorescence quenching. Subsequently, the potential Q-markers of GSB linked to the treatment of liver injury were verified using an *in vitro* cell assay. Finally, the quality of GSB samples from different producing areas was evaluated using chemical pattern recognition methods. This study provides a foundation for improving the quality standard of GSB and promoting its clinical application, as well as offering a new approach for TCM Q-marker research.

## 2 Materials and methods

### 2.1 Instruments and materials

The following equipment was used in this study: An Agilent 1260 high-performance liquid chromatograph (Agilent, America); a high-speed universal crusher (Test Instrument, Tianjin, China); a DHG-9145A air-drying oven (Yiheng Scientific Instrument, Shanghai, China); a PT-35SL micro electronic balance (Huazhi Electronic Technology, Putian, Fujian, China); KQ3200DM silent and KQ5200DB numerical control ultrasonic cleaners (Ultrasonic Instrument, Kunshan, Jiangsu, China); SHZ-D (III) Multipurpose Circulating Water Vacuum Pump (Yuhua Instrument, Zhengzhou, Henan, China); WKY II2 micropipette (Jia’an Analytical Instrument Factory, Shanghai, China); SpectraMax i3X multifunction spectrometer (Megu Molecular Instruments, Shanghai, China); SRFSTPRP-CL freezing grinder (Sheyan Instrument, Shanghai, China); TGL-16M centrifuge (Xiangyi Laboratory Instrument Development, Changsha, Hunan, China); HF-240 CO_2_ incubator and HFLTP 86 ultra-low temperature refrigerator (Lixin Instrument, Shanghai, China); Nexcope inverted microscope (Yongxin Optics, Ningbo, Zhejiang, China); and UV-5200 ultraviolet-visible spectrophotometer (Yuanxi Analysis Instrument, Shanghai, China).

Loganic acid, swertiamarin, gentiopicroside, and sweroside standards were supplied by Solarbio (Beijing, China). The *6*′-*O*-*β*-*D*-glucosylgentiopicroside standard was provided by Yuanye Biotechnology (Shanghai, China). The purity of all the above standards was greater than 98%. Chromatography-grade methanol was purchased from J.T. Baker Chemical Company (Shanghai, China). Purified water was bought from Wahaha Group (Hangzhou, Zhejiang, China).

Horse serum and trypsin were purchased from Thermo Fisher (Shanghai, China). Penicillin-Streptomycin-Amphotericin B Solution (100×), non-essential amino acids, high-glucose DMEM, dimethyl sulfoxide (DMSO), high-efficiency RIPA lysis buffer, BCA protein assay kit, protease and phosphatase inhibitor mixture, Dulbecco’s phosphate-buffered saline (D-PBS), Cell Counting Kit-8 (CCK-8), alanine aminotransferase (ALT), and aspartate aminotransferase (AST) detection kits were supplied by Solarbio (Beijing, China). BCA protein assay kit; Western and IP cell lysis buffer; and phenylmethylsulfonyl fluoride (PMSF), malondialdehyde (MDA), tumor necrosis factor-alpha (TNF-α), interleukin-1 beta (IL-1β), and IL-6 detection kits were bought from Beyotime (Shanghai, China). Total antioxidant capacity (T-AOC), superoxide dismutase (SOD), lactate dehydrogenase (LDH), glutathione (GSH), and catalase (CAT) determination kits were procured from Nanjing Jiacheng (Nanjing, Jiangsu, China).

A total of 44 batches of wild and cultivated GSB were collected from Liaoning, Jilin, Heilongjiang, and Inner Mongolia, with sample numbers S1 to S44, respectively. Specific sample information is provided in Table 1. The samples were identified as GSB by Prof. Haibo Yin (Liaoning University of Traditional Chinese Medicine). GSB roots and rhizomes were ground using a grinder and passed through an 80-mesh sieve to obtain GSB dried powder.

**TABLE 1:** Source information of GSB.

Sample no.	Place of collection	Type	Sample no.	Place of collection	Type
S1	Liaoning, China	Wild	S23	Liaoning, China	Cultivated
S2	Liaoning, China	Wild	S24	Liaoning, China	Cultivated
S3	Liaoning, China	Wild	S25	Liaoning, China	Cultivated
S4	Liaoning, China	Wild	S26	Liaoning, China	Cultivated
S5	Liaoning, China	Wild	S27	Liaoning, China	Cultivated
S6	Liaoning, China	Wild	S28	Liaoning, China	Cultivated
S7	Liaoning, China	Wild	S29	Liaoning, China	Cultivated
S8	Liaoning, China	Wild	S30	Liaoning, China	Cultivated
S9	Liaoning, China	Wild	S31	Liaoning, China	Cultivated
S10	Liaoning, China	Wild	S32	Liaoning, China	Cultivated
S11	Liaoning, China	Wild	S33	Liaoning, China	Cultivated
S12	Liaoning, China	Wild	S34	Liaoning, China	Cultivated
S13	Liaoning, China	Wild	S35	Liaoning, China	Cultivated
S14	Liaoning, China	Wild	S36	Liaoning, China	Cultivated
S15	Jilin, China	Wild	S37	Liaoning, China	Cultivated
S16	Jilin, China	Wild	S38	Liaoning, China	Cultivated
S17	Jilin, China	Wild	S39	Liaoning, China	Cultivated
S18	Jilin, China	Wild	S40	Liaoning, China	Cultivated
S19	Heilongjiang, China	Wild	S41	Liaoning, China	Cultivated
S20	Nei Mongol, China	Wild	S42	Liaoning, China	Cultivated
S21	Nei Mongol, China	Wild	S43	Liaoning, China	Cultivated
S22	Jilin, China	Cultivated	S44	Liaoning, China	Cultivated

The liver total protein used in this experiment was extracted from fresh mouse liver tissue (species: ICR mice, 6–8 weeks old, male; provided by the Experimental Animal Center of Liaoning University of Traditional Chinese Medicine, license number: SCXK (Liao) 2020-0001). NCTC 1469 mouse liver cells were bought from iCell Bioscience Inc. (Shanghai, China) and were maintained in an incubator at 37 °C with 5% CO_2_.

### 2.2 HPLC fingerprint analysis

#### 2.2.1 Preparation of sample and standard solution

The appropriate amount of each standard was accurately weighed and dissolved in methanol to prepare a standard solution containing 1 mg/mL of each component (loganic acid, *6*′-*O*-*β*-*D*-glucosylgentiopicroside, swertiamarin, gentiopicroside, and sweroside). After shaking well, the solution was filtered through a 0.22-μm hydrophilic microfiltration membrane, and the filtrate was collected and stored at 4 °C for later use.

For each of the 44 batches, 0.5 g of GSB powder was accurately weighed and placed in a 50-mL stoppered conical flask. Subsequently, 20 mL of methanol was added, and the flask was weighed, followed by ultrasonic extraction (25 °C, 200 W, 30 min). The extract was then cooled to room temperature, reweighed, and the lost weight was compensated for with methanol. After thorough shaking, the mixture was filtered, and the resulting filtrate was passed through a 0.22-μm hydrophilic microfiltration membrane, yielding the test solution.

#### 2.2.2 Fingerprint conditions

A Waters Symmetry Column (250 mm × 4.6 mm, 5 μm) (Waters Corporation, United States) was used for chromatographic separation. The mobile phase consisted of methanol (solvent A) and water: phosphoric acid solution (1,000:2, v/v; solvent B). The following gradient elution program was used: 0–20 min, 8%–32% A; 20–45 min, 32%–60% A; 45–80 min, 60%–95% A. The column temperature was set at 30 °C, the flow rate was 0.8 mL/min, the detection wavelength was 240 nm, and the injection volume was 10 μL.

#### 2.2.3 Method validation

Precision was verified by performing six consecutive injections of the same sample solution. Repeatability was evaluated by independently preparing six parallel sample solutions. Stability was assessed by repeatedly analyzing the same sample at 0, 2, 4, 8, 12, and 24 h using the above-described chromatographic method. The relative standard deviation (RSD) of each characteristic peak area and retention time was calculated to evaluate the overall precision.

#### 2.2.4 Establishment and similarity evaluation of the chromatographic fingerprint

The Similarity Evaluation System for TCM Chromatographic Fingerprint (2012 edition; Beijing, China) was employed for data analysis. The HPLC chromatograms of GSB samples from the different producing areas were calibrated at multiple points, and chromatographic peaks were matched at a time width of 0.10 min. A reference chromatogram (R) was generated using the median method, and similarity was subsequently calculated. Based on these results, the characteristic chromatographic fingerprints for GSB from different producing areas were established.

#### 2.2.5 Fingerprint analysis

Principal Component Analysis (PCA) is an unsupervised dimensionality reduction technique that retains the maximum amount of original data variance, while describing the characteristics of the original data using a reduced set of new variables (Zeng et al., 2021). Here, PCA was conducted on GSB samples from different producing regions based on eigenvalues and cumulative contribution rates. Subsequently, Orthogonal Partial Least Squares Discriminant Analysis (OPLS-DA) was applied to the areas of 25 common peaks to clarify quality differences among GSB samples of different origins. To identify the main differential variables between sample groups, the Variable Importance in Projection (VIP) method was employed, with a VIP value greater than 1 serving as the significance standard. A larger VIP value indicates a greater contribution of that component to the observed group differences.

### 2.3 Analysis of “small molecule–protein” interactions

#### 2.3.1 Protein preparation

Fresh mouse liver tissue (0.5 g) was rinsed with D-PBS to remove blood, cut into small pieces, and mixed with 1.5 mL of pre-cooled high-efficiency RIPA lysis buffer containing 1% PMSF and a 1% protease/phosphatase inhibitor mixture. Subsequently, the mixture was subjected to ultrasonic lysis on ice (power: 300 W, working time: 3 s, interval time: 5 s, total cycles: 20) to fully lyse the cell membrane. The lysate was then centrifuged at 12,000 × g for 15 min at 4 °C, and the supernatant (containing total liver protein) was collected.

#### 2.3.2 Condition selection

A single-factor method was used to determine the reaction duration. Using 1 mg/mL liver protein and 1 mg/mL gentiopicroside as the model system, the mixture was incubated for 10, 20, 30, 40, 50, 60, 70, and 80 min. The fluorescence intensity was measured at the 360 and 460 nm excitation and emission wavelengths, respectively, to identify the optimal incubation duration.

To simplify the experiment, the protein mass concentration was uniformly set at 1 mg/mL, and reference solutions with mass concentrations of 0.1, 0.2, 0.4, 0.6, 0.8, and 1.0 mg/mL were prepared. These solutions were allowed to react with the liver protein solution, and the optimal reaction concentration of the small-molecule components was determined by monitoring changes in fluorescence intensity.

### 2.4 Detection of the interactions between the small molecules and liver total protein

DMEM solutions each containing one of five reference products (each at 1 mg/mL) were mixed with the liver total protein solution (1 mg/mL), and the mixture was transferred to a 96-well microplate (black background and lid). After incubation at 37 °C for 10 min, the following four control groups were set up: A: 200 μL of lysate; B: 200 μL of lysate + 10 μL of 1 mg/mL reference solution; C: 200 μL of liver protein solution; and D: 200 μL of liver protein solution +10 μL of 1 mg/mL reference solution. Each experiment was performed in triplicate with three technical replicates per condition.

### 2.5 Determination of the optimal conditions for establishing the H_2_O_2_-induced hepatocyte damage model

NCTC 1469 cells (180 μL) in the logarithmic growth phase were inoculated in 96-well plates at a density of 2 × 10^5^ cells/mL and cultured for 24 h at 37 °C under 5% CO_2_. Then, 20 μL of H_2_O_2_ solutions at different concentrations (1,000, 1,100, 1,200, 1,300, 1,400, 1,500, and 1,600 μmol/L) was added to the cells for 0, 2, 4, 6, 8, 10, and 12 h. After treatment, the medium was removed, and the plate was washed twice with PBS. Subsequently, 100 μL of fresh medium and 10 μL of CCK-8 reagent were added, and the cells were cultured for 2.5 h. Absorbance at 450 nm was measured to determine the optimal H_2_O_2_ concentration and exposure time required to establish the liver cell damage model.

### 2.6 Cytotoxicity assay

NCTC 1469 cells (180 μL) in the logarithmic growth phase were inoculated in 96-well plates at a density of 2 × 10^5^ cells/mL and cultured under 5% CO_2_ at 37 °C for 24 h. Then, 20 μL of swertiamarin, gentiopicroside, or sweroside solution at different concentrations (6.25, 12.5, 25, 50, 100, 200, and 400 μmol/L) was added to the cells. After 12 h of treatment, the medium was removed, and the plate was washed twice with D-PBS. Subsequently, 100 μL of fresh medium and 10 μL of CCK-8 reagent were added, and the cells were cultured for 2.5 h. Absorbance at 450 nm was measured to determine the optimal dose concentration and treatment duration.

### 2.7 Index measurement

NCTC 1469 cells (2 × 10^6^/mL) were seeded in 6-well plates (1.8 mL per well) and cultured in an incubator (37 °C, 5% CO_2_) for 24 h. Hepatocyte injury was then induced by adding 0.2 mL of H2O2 solution to each well using the optimal H_2_O_2_ concentration and treatment duration determined in Section 2.6. The medium was then aspirated, the plate was washed twice with D-PBS, and 0.2 mL of fresh medium containing swertiamarin, gentiopicroside, or sweroside at low, medium, and high concentrations (6.25, 12.5, and 25 μmol/L, respectively) was added to the cells. After 24 h of culture, the T-AOC and the levels of MDA, SOD, CAT, GSH, ALT, AST, and the inflammatory factors TNF-α, IL-1β, and IL-6 were determined for each group.

### 2.8 Content determination

#### 2.8.1 Preparation of sample and standard solution

The methods used for the preparation of reference and test sample solutions were as described in Section 2.2.1.

#### 2.8.2 Conditions used for content determination

A Waters Symmetry Column (250 mm × 4.6 mm, 5 μm) was used for chromatographic separation. The mobile phase consisted of methanol (solvent A) and water: phosphoric acid (1000:2, v/v; solvent B). The following gradient elution program was used: 0–5 min, 20%–30% A; 5–12 min, 30% A; 12–18 min, 30%–36% A. The column temperature was set at 30 °C, the flow rate was 0.8 mL/min, the detection wavelength was 240 nm, and the injection volume was 5 μL.

#### 2.8.3 Validation of the method used for HPLC quantification

Method accuracy, repeatability, and stability were verified by calculating the RSD of the peak areas of swertiamarin, gentiopicroside, and sweroside, as described in Section 2.2.3. Calibration curves for these three compounds were plotted based on a linear regression analysis of the integrated peak areas (mAU) *versus* concentration (mg/mL). For the spike recovery test, six portions of powdered GSB medicinal material with known content were accurately weighed (0.50 g). Appropriate amounts of the respective swertiamarin, gentiopicroside, and sweroside reference substances were then added to each portion. The test solution was prepared according to the method described in Section 2.2.1, and analyzed under the chromatographic conditions specified in Section 2.8.2. The spike recovery rate was subsequently calculated.

### 2.9 Statistical analysis

The activity percentage in the small molecule–protein interaction detection experiments was calculated using the following equation: Activity Percentage = [(the combined fluorescence intensities of total protein and control products − the fluorescence intensity of the incubation system)/(the combined fluorescence intensities of total protein and control products)] × 100% [(FB + FC − 2FA − (FD − FA))/(FB + FC − 2FA) × 100%] (where FD−FA represent the fluorescence intensity of controls A to D). All biological activity-related data were analyzed using GraphPad Prism 8.0 (GraphPad Software, CA, United States), SIMCA (version 14.1, Umetrics, SE), and are presented as means ± standard deviation (SD). One-way analysis of variance (ANOVA) was performed for multiple comparisons, with statistical significance set at P < 0.05. All relational analyses were performed using SPSS (version 26.0, IBM, United States).

## 3 Results

### 3.1 Fingerprinting

#### 3.1.1 Reference peak selection

The peak of gentiopicroside in the chromatograms of GSB of differing origins was well-separated, with a stable peak shape and a large peak area. As it was present in all samples, the gentiopicroside peak was designated as the reference peak (S).

#### 3.1.2 Method validation

In the evaluation of the precision, stability, and repeatability of the HPLC fingerprint method, the RSD values for the relative peak areas and relative retention times of the 44 batches of GSB from different origins were all below 3%.

#### 3.1.3 Fingerprint establishment and similarity evaluation

**FIGURE 1:**
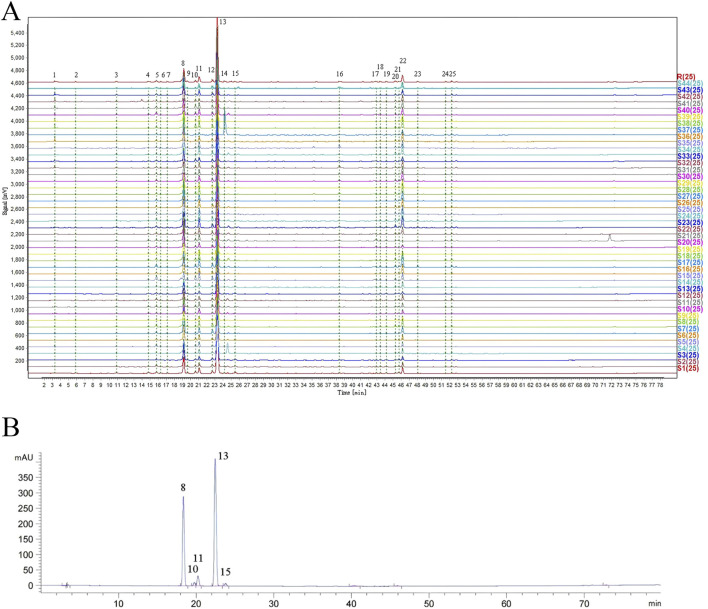
HPLC fingerprint of 44 GSB samples **(A)** Mixed control HPLC fingerprint **(B)**.

A total of 25 common peaks were identified among the 44 batches of GSB. The relative peak areas and relative retention times of the 25 peaks are detailed in Supplementary Tables S1 and S2. The RSD of the relative retention times of the common fingerprint peaks displayed little variation, ranging from 0.635% to 3.917%. In contrast, the RSDs for the relative peak areas were significantly different, with values between 19.439% and 144.854% (Supplementary Tables S3 and S4). Based on reference standards, peaks 8, 10, 11, 13, and 15 were identified as loganic acid, *6*′-*O*-*β*-*D*-glucosylgentiopicroside, swertiamarin, gentiopicroside, and sweroside, respectively. The chromatographic superposition is shown in Figure 1A, and the mixed standard comparison is shown in Figure 1B. Similarity was found to range from 0.958 to 1.000, suggesting that their chemical characteristics were highly similar. The similarity evaluation results are shown in Table 2.

**TABLE 2:** Similarity evaluation results of GSB samples.

Sample no.	Similarity	Sample no.	Similarity	Sample no.	Similarity	Sample no.	Similarity
S1	0.996	S12	0.984	S23	0.999	S34	0.993
S2	1.000	S13	0.998	S24	1.000	S35	0.958
S3	0.999	S14	0.989	S25	0.994	S36	1.000
S4	0.993	S15	0.999	S26	0.997	S37	0.999
S5	0.997	S16	0.999	S27	1.000	S38	0.999
S6	0.989	S17	0.999	S28	0.999	S39	0.999
S7	0.996	S18	0.997	S29	0.996	S40	0.998
S8	0.999	S19	0.996	S30	0.996	S41	0.999
S9	0.983	S20	0.992	S31	0.989	S42	0.999
S10	1.000	S21	0.998	S32	0.995	S43	0.996
S11	0.998	S22	0.993	S33	0.999	S44	0.994

#### 3.1.4 PCA and OPLS-DA

**FIGURE 2:**
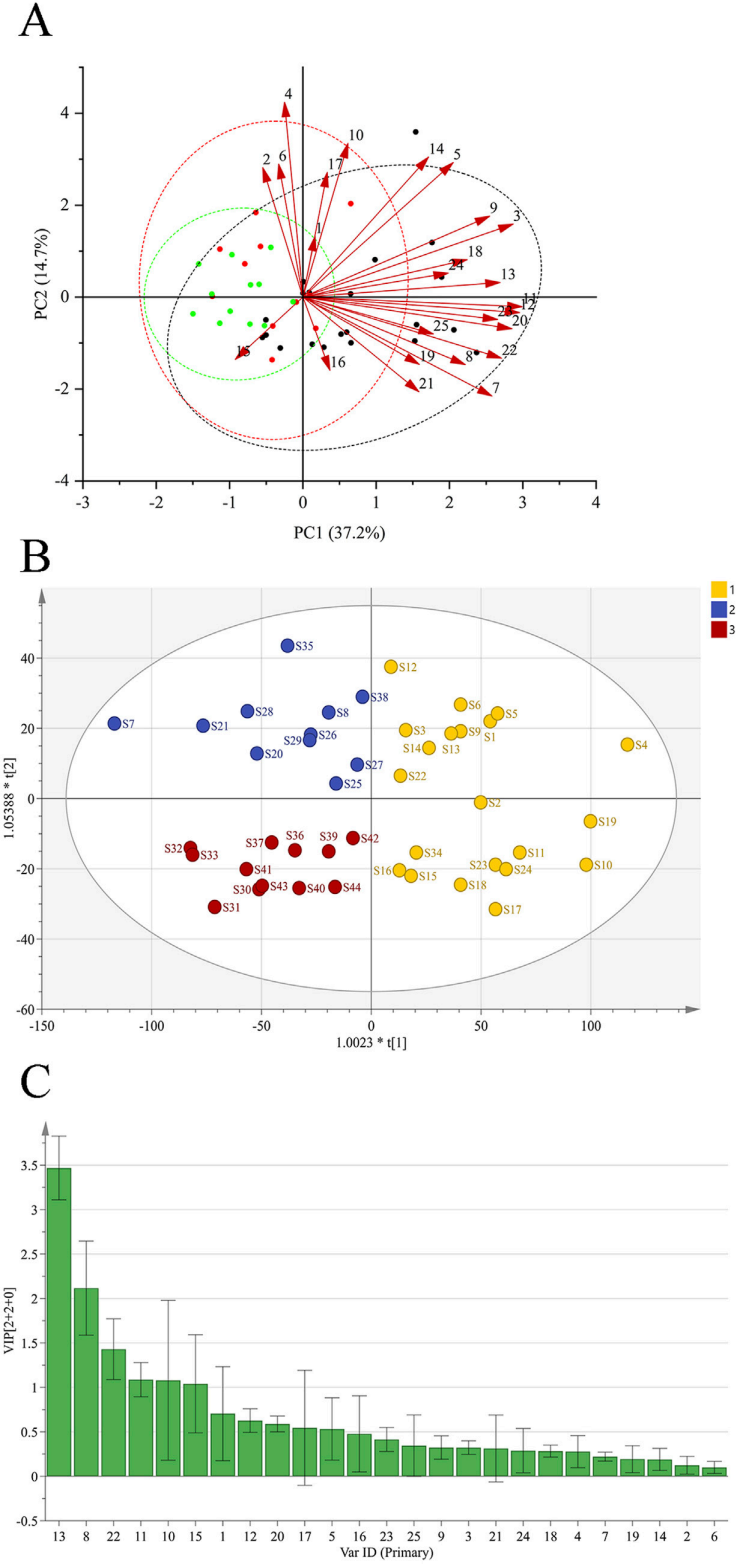
PCA of GSB from different origins **(A)** OPLS-DA scores of GSB from different origins **(B)** Importance variables of GSB from different origins **(C)**.

A total of six principal components (PCs) were extracted in the PCA. As shown in Table 3, the cumulative contribution of the first three PCs reached 85.47%, indicating that these three PCs effectively captured the main information of the samples. The eigenvalue of PC1 was 9.920, with a variance contribution rate of 39.680%. For PC2, the eigenvalue was 3.536, and the variance contribution rate was 14.143%. PC3 exhibited an eigenvalue of 2.787, with a corresponding variance contribution rate of 11.148%. The factor loading matrix for the common peaks in each PC is shown in Table 4. The resulting PCA plot (Figure 2A) shows that the 44 GSB batches clustered into three distinct groups. The arrow length and the angle between the coordinate axes represent the contribution of each common peak area to the corresponding PC.

**TABLE 3:** GSB PCA eigenvalues and variance contribution rate.

Ingredients	Initial eigenvalues			Principal factor contribution rate		
	Total	Variance percentage	Variance cumulative	Total	Variance percentage	Variance cumulative
1	9.92	39.680	39.68	9.92	39.68	39.68
2	3.536	14.143	53.823	3.536	14.143	53.823
3	2.787	11.148	64.971	2.787	11.148	64.971
4	1.813	7.250	72.221	1.813	7.25	72.221
5	1.243	4.971	77.192	1.243	4.971	77.192
6	1.119	4.478	81.67	1.119	4.478	81.67

**TABLE 4:** Principal component factor loading matrix for common peaks in GSB samples.

Commom peak numbers	PCA1	PCA2	PCA3	PCA4	PCA5	PCA6
1	0.097	0.205	−0.757	−0.006	0.080	0.239
2	0.057	0.630	0.569	0.014	0.059	0.051
3	0.912	0.247	0.023	−0.040	0.096	0.004
4	0.107	0.848	0.266	−0.139	−0.013	−0.071
5	0.699	0.479	−0.090	0.379	−0.156	−0.070
6	0.118	0.620	0.305	−0.061	0.154	0.464
7	0.781	−0.435	−0.025	−0.207	0.168	−0.168
8	0.699	−0.282	0.084	−0.405	0.291	−0.260
9	0.829	0.280	0.148	0.221	−0.165	−0.075
10	0.262	0.593	−0.404	−0.001	−0.130	−0.271
11	0.919	−0.090	0.000	0.136	0.063	−0.071
12	0.916	−0.077	0.051	0.127	0.090	−0.032
13	0.864	0.062	0.335	−0.014	0.165	−0.058
14	0.584	0.531	0.080	−0.190	0.107	−0.181
15	0.145	−0.280	0.041	0.492	0.656	0.280
16	−0.168	−0.120	0.720	0.445	−0.054	0.257
17	0.159	0.482	−0.475	−0.127	0.307	0.313
18	0.707	0.084	−0.077	0.429	−0.365	−0.042
19	0.548	−0.224	0.516	−0.333	0.013	−0.012
20	0.864	−0.200	−0.154	0.175	0.173	−0.110
21	0.533	−0.343	0.439	−0.222	−0.294	0.213
22	0.831	−0.296	−0.174	0.207	−0.097	0.115
23	0.789	−0.193	−0.311	0.205	−0.146	0.153
24	0.630	0.045	−0.111	−0.548	0.021	0.255

An OPLS-DA score plot (Figure 2B) and a VIP plot (Figure 2C) were generated, resulting in the selection of six variables with high contribution rates for group differentiation. Based on reference standards, peaks 8, 10, 11, 13, and 15 corresponded to loganic acid, *6*′-*O*-*β*-*D*-glucosylgentiopicroside, swertiamarin, gentiopicroside, and sweroside, respectively. While the VIP value for peak 22 was also greater than 1, the component could not be identified, as liquid chromatography-mass spectrometry (LC-MS) was not performed in this study, a limitation that will be addressed in subsequent work. These five components thus likely play an important role in distinguishing GSB samples based on their origin, and are also likely to be the main characteristic components of GSB.

### 3.2 Small molecule–protein interactions

#### 3.2.1 Condition optimization

Incubation temperature, pH, and duration can all influence experimental small molecule–protein interactions (Xu, 2013). To simulate physiologically relevant conditions, the temperature was set to 37 °C and the pH was maintained at 7.0, with incubation time being further optimized. Furthermore, proteins from different tissues may have different fluorescence characteristics, while elevated small-molecule or protein concentrations can result in a self-quenching phenomenon (Yang et al., 2007). Consequently, the excitation and emission wavelengths, small-molecule chemical compositions, and protein concentrations in the solution were optimized.

According to the literature, the stable excitation-emission wavelength range for liver protein is 360–450 nm. This wavelength range was chosen for the experiment (Hui et al., 2023) because preliminary evaluation indicated that it effectively eliminated component autofluorescence interference, thereby ensuring the accuracy of interaction detection. Moreover, as shown in Supplementary Table S5, the interaction intensity between liver protein and gentiopicroside remained stable across an incubation duration of 10–80 min. Consequently, the incubation time was set at 10 min. Meanwhile, it was observed that as the small-molecule compound concentration increased, the fluorescence intensity during the interaction with liver protein gradually decreased. Therefore, a 1 mg/mL small-molecule compound solution was selected for the interaction with liver protein (Supplementary Table S6).

The specific detection parameters were slit width: excitation = 5 nm, emission = 5 nm; gain = 700 V; scanning speed = 100 nm/min.

#### 3.2.2 Detection of component activity and Q-marker screening

To evaluate the potential biological activity of the pharmaceutical components, the interactions between five chemical constituents and liver proteins were measured, and their corresponding activity percentages were determined. Given that the absolute values of the original activity percentages varied significantly among the five components, Z-score normalization [Z = (sample value −mean)/SD] was applied to eliminate dimensional effects, thus facilitating comparisons of relative activities. The effects of these five components on liver proteins were found to vary, with swertiamarin, gentiopicroside, and sweroside exhibiting the most significant effects, as summarized in Supplementary Table S7. Based on the principle of component effectiveness, these three components were ultimately selected as the active Q-markers of GSB.

### 3.3 The optimal H_2_O_2_ concentration and exposure duration for inducing oxidative damage in hepatocytes

**FIGURE 3:**
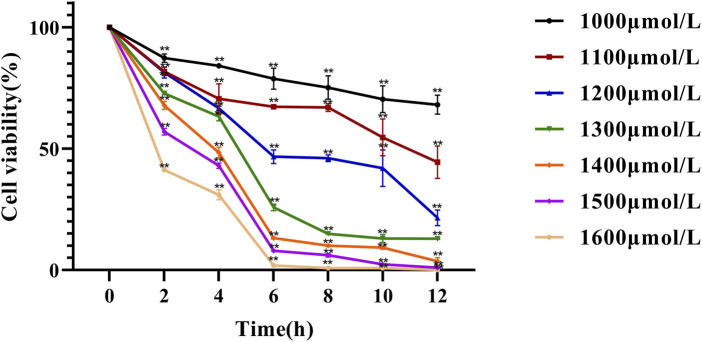
Effect of hydrogen peroxide on NCTC-1469 cell viability line chart. Data are shown as mean ± SD (*n* = 6), * *p* < 0.05, ** *p* < 0.01.

In this experiment, NCTC 1469 cells were exposed to different concentrations of H2O2 for different durations, and cell viability was determined using the CCK-8 assay (Figure 3). The results showed that both cell number and viability significantly decreased with increasing H_2_O_2_ concentrations and treatment durations. At low concentrations, H2O2 induced only mild damage to liver cells, and thus could not achieve the experimental objective, whereas excessively high concentrations caused extensive liver cell death. Meanwhile, a concentration of 1300 μmol/L and a 4 h treatment time resulted in an approximately 30% inhibition of cell viability compared with controls (*P* < 0.01). Therefore, 1,300 μmol/L and 4 h were selected as the optimal H2O2 concentration and treatment duration for inducing hepatocyte injury in subsequent experiments. These parameters are highly consistent with those commonly reported in the literature (Wei Y. et al., 2021; Wang et al., 2023).

### 3.4 The cytotoxicity of swertiamarin, gentiopicroside, and sweroside

**FIGURE 4:**
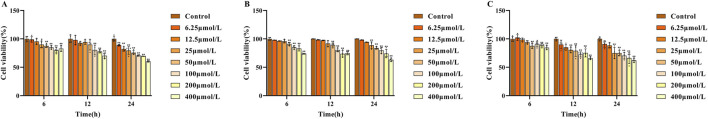
A: Effect of different concentrations of swertiamarin **(A)**, gentiopicroside **(B)** sweroside **(C)** on the viability of NCTC-1469 cells.

For evaluating the cytotoxicity of the three potential Q-markers, NCTC 1469 cells were treated with different concentrations of each compound for 6, 12, and 24 h, and cell viability was subsequently determined using the CCK-8 assay (Figures 4A–C). The results showed that cell viability was suppressed to varying degrees with increasing drug concentrations and treatment times. At 6 h, cell viability remained >90% at concentrations of 6.25, 12.5, and 25 μmol/L for swertiamarin, and 6.25, 12.5, 25, and 50 μmol/L for both gentiopicroside and sweroside. At 12 h, cell viability exceeded 90% at 6.25, 12.5, and 25 μmol/L for all three compounds. However, at 24 h, swertiamarin treatment reduced cell viability to below 90% with all tested concentrations, while cell viability remained above 90% at the concentrations of 6.25 and 12.5 μmol/L for gentiopicroside and 6.25 μmol/L for sweroside. To ensure therapeutic efficacy, the concentrations of 6.25, 12.5, and 25 μmol/L (low, medium, and high) were selected for investigating their antioxidant effects on damaged hepatocytes.

### 3.5 The activity of MDA, SOD, and LDH, and assessment of T-AOC

**FIGURE 5:**
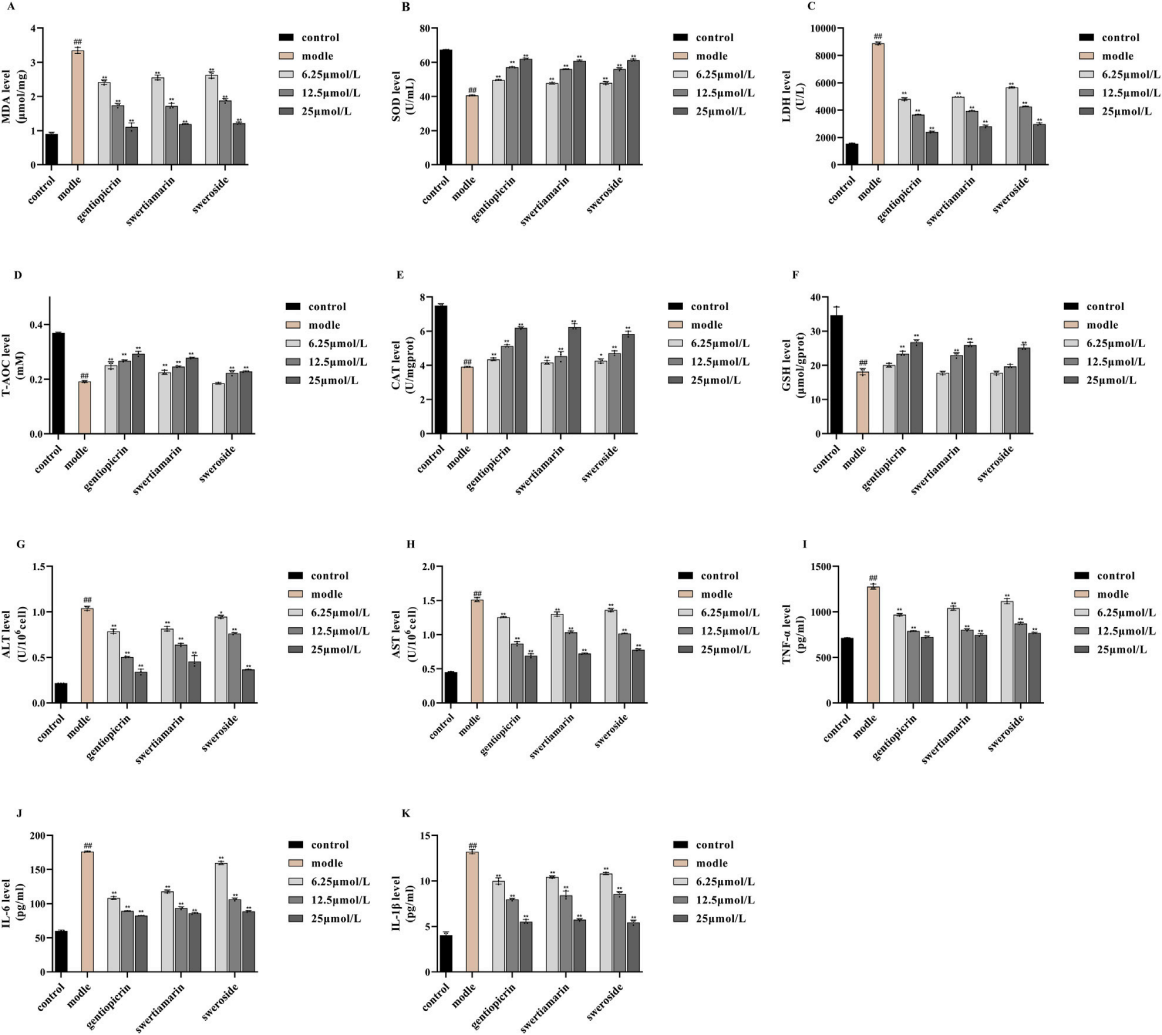
Effects of swertiamarin, gentiopicroside, and sweroside on the activities of MDA **(A)**, SOD **(B)**, LDH **(C)** and T-AOC **(D)**,CAT **(E)**, GSH **(F)**, ALT **(G)**, AST **(H)**, TNF-α **(I)**, IL-1β **(J)**, IL-6 **(K)** in NCTC-1469 cells. Results are expressed as mean ± SD (n = 6). ^##^ *p* < 0.01 compared with the control group; ^**^ *p* < 0.01 compared to model group.

Free radicals generated from normal substances, drugs, or poisons during metabolic processes can induce lipid peroxidation. MDA, a product of lipid peroxidation, damages biological macromolecules, thereby exacerbating cell membrane injury. Consequently, the MDA concentration reflects the degree of cellular lipid peroxidation, indirectly indicating the extent of cell damage (Finkel and Oxidants, 2000). SOD and LDH serve as key indicators of cellular free radical metabolism and play a crucial role in maintaining the oxidant-antioxidant balance. Their levels indirectly reflect the body’s free radical scavenging capacity and overall bodily damage (Djordjevic et al., 2004). T-AOC refers to the combined antioxidant potential of various antioxidant molecules, including bioactive compounds (Silvestrini et al., 2023). Here, the levels of MDA, SOD, and LDH, along with the T-AOC, served as indices for evaluating the oxidative stress response in NCTC 1469 cells. As shown in Figures 5A–D, compared with controls, MDA and LDH levels in NCTC 1469 cells were significantly increased after 4 h of treatment with 1300 μmol/L H_2_O_2_, while SOD activity and T-AOC exhibited a marked decline. These results indicated that H_2_O_2_ treatment severely damaged NCTC 1469 cells.

Notably, compared with the model group, the addition of swertiamarin, gentiopicroside, or sweroside led to reductions in both MDA production and LDH release, indicating that all three compounds suppress lipid peroxidation and alleviate oxidative stress-induced damage in hepatocytes. Concomitantly, SOD activity and the T-AOC were significantly increased in the treatment groups, suggesting that these compounds restored free radical scavenging ability in injured hepatocytes and enhanced their T-AOC. For all three compounds, the most pronounced therapeutic effect on hepatocyte injury was observed with the 25 μmol/L concentration.

### 3.6 CAT and GSH activities

Under conditions of cellular aging or stress, ROS metabolism increases, leading to H2O2 accumulation and, consequently, cell damage. CAT is a key enzyme in the antioxidant defense system. Its main function is to catalyze the decomposition of H_2_O_2_ into water and oxygen, thus protecting cells from H_2_O_2_-induced toxicity (Zhang and Tian, 2007). GSH is the body’s most important non-enzymatic antioxidant, performing essential physiological functions, including scavenging free radicals, detoxifying harmful substances, promoting iron absorption, maintaining normal cell growth and development, and modulating immune responses (Pinho et al., 2006; Korkmaz et al., 2010). Accordingly, in this study, the levels of CAT and GSH served as indices for evaluating the oxidative stress response in NCTC 1469 cells. As shown in Figures 5E,F, in hepatocytes exposed to 1,300 μmol/L H_2_O_2_ for 4 h, CAT and GSH levels were reduced to differing extents compared with those in model cells. Importantly, following the addition of swertiamarin, gentiopicroside, or sweroside, hepatocyte activity was restored, accompanied by an increase in CAT and GSH contents. These results indicated that swertiamarin, gentiopicroside, and sweroside mitigate H2O2-induced hepatocyte damage and enhance the antioxidant capacity of injured hepatocytes. Again, for all three compounds, the 25 μmol/L concentration exerted the most significant therapeutic effect on hepatocyte injury.

### 3.7 ALT and AST activities

ALT is predominantly found in liver cells, where its intracellular concentrations are 1,000–3,000 times higher than those in serum. However, damage to just 1% of liver cells can lead to the doubling of serum ALT levels. The World Health Organization recognizes ALT as the most sensitive marker for assessing liver function injury (Cheng, 2017). AST is mainly found in mitochondria, with the highest concentration detected in cardiomyocytes, followed by hepatocytes. Damage to cardiomyocytes or hepatic mitochondria results in AST leaking into the bloodstream, thus elevating its levels in serum. Consequently, serum AST represents a sensitive readout of the degree of damage to cardiac and hepatic cells (Dembek et al., 2017).

In our study, the levels of ALT and AST were used as indicators for assessing the extent of NCTC 1469 cell damage. We found that compared with controls, ALT and AST levels were significantly increased in NCTC 1469 cells after treatment with 1300 μmol/L H_2_O_2_ for 4 h, indicating that these cells had experienced notable damage. Nevertheless, treatment with swertiamarin, gentiopicroside, or sweroside significantly reduced the H_2_O_2_-induced increases in ALT and AST release (Figures 5G,H). These results implied that all three GSB compounds mitigated H_2_O_2_-induced hepatocyte damage, with 25 μmol/L eliciting superior therapeutic efficacy.

### 3.8 The levels of inflammatory cytokines (TNF-α, IL-6, and IL-1β)

TNF-α functions as a key mediator in the regulation of inflammation, cell proliferation, apoptosis, and immunity. It has been reported to induce hepatocyte apoptosis and promote hepatocellular carcinoma cell proliferation through distinct mechanisms, indirectly contributing to liver cancer development associated with fatty liver disease and chronic viral hepatitis, as well as promoting liver cancer progression. Notably, TNF-α is regarded as playing a key pro-inflammatory role in the pathophysiology of liver disease (Chen et al., 2019; Jang et al., 2014). IL-1β exhibits potent pro-inflammatory activity and induces the production of a variety of pro-inflammatory mediators (Malkova et al., 2023). Studies have indicated that IL-1β is involved in inflammatory responses across most acute and chronic liver diseases, playing a pivotal role in their progression (Huai and Wang, 2023). IL-6 also possesses pro-inflammatory properties, inducing the production of acute phase proteins, causing liver damage, and promoting liver aging, fibrosis, steatosis, and carcinogenesis (Wang et al., 2024). Therefore, in this study, the levels of these inflammatory factors—TNF-α, IL-6, and IL-1β—served as indicators of NCTC 1469 cell damage. As shown in Figures 5I–K, compared with the control condition, treatment with 1300 μmol/L H2O2 significantly increased the contents of the three proinflammatory factors in NCTC 1469 cells. Importantly, the levels of TNF-α, IL-6, and IL-1β decreased after the addition of swertiamarin, gentiopicroside, or sweroside, indicating that these compounds mitigated H2O2-induced inflammatory responses in hepatocytes. As observed for the other markers of liver damage, the best anti-inflammatory effects of the GSB Q-markers were observed with the 25 μmol/L concentration.

### 3.9 Quantitative analysis

The regression equations of swertiamarin, gentiopicroside, and sweroside were defined as *y* = 4,650.7*x* + 1.0924, *y* = 5,908.2*x* + 61.304, and *y* = 8,083.9*x* − 1.5587, respectively. The RSD values for the peak areas from precision, stability, and repeatability experiments ranged from 0.85% to 3.49%, while the RSD values for the relative retention times from these experiments varied between 0.04% and 1.20%, all below 4%. Furthermore, the average spike recovery rates of each component were in the range of 98.82%–100.37%, and all RSD values were between 1.00% and 2.70%.

**FIGURE 6:**
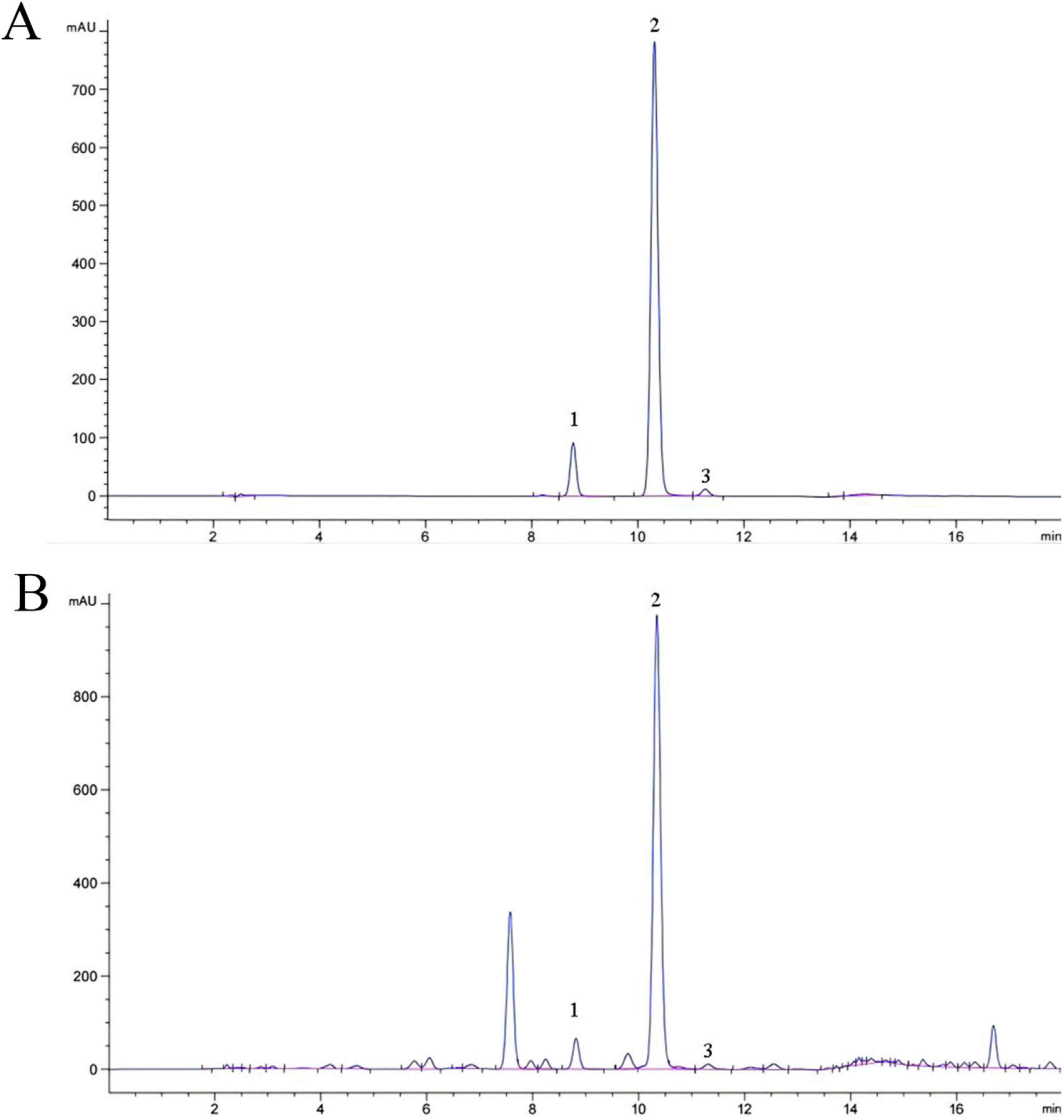
HPLC chromatograms of reference **(A)** and sample **(B)**.

The chromatogram of the mixed reference standard and sample obtained under the chromatographic conditions described in Section 2.8.1 is shown in Figures 6A,B. The original peak areas of the three components (swertiamarin, gentiopicroside, and sweroside) in the 44 GSB samples from different producing areas are shown in Supplementary Table S8, and the calculated content results are presented in Table 5. The content of swertiamarin was highest in S23 and lowest in S42. The highest and lowest contents of gentiopicroside were recorded in S10 and S42, respectively. The highest sweroside content was observed in S9, and the lowest in S33. There were significant differences in the total contents of the three Q-markers across the GSB samples from the different growing regions. The highest total content of the three components, 86.1722 mg/g, was noted in S10, while the lowest was seen in S42, totaling only 32.1386 mg/g. These findings indicated that growing conditions influence the contents of chemical components in GSB.

**TABLE 5:** Results of Q*-*markers content (mg/g, *n* = 3).

NO.	Swertiamarin	Gentiopicroside	Sweroside	Total content	NO.	Swertiamarin	Gentiopicroside	Sweroside	Total content
S1	6.9970	73.4831	0.7078	81.1879	S23	8.3188	65.7163	0.7930	74.8281
S2	5.0636	70.5988	0.8001	76.4625	S24	5.8430	65.4221	0.7822	72.0473
S3	4.1179	68.1956	0.7008	73.0142	S25	4.5059	52.1401	0.5083	57.1543
S4	7.7202	66.7382	0.7201	75.1785	S26	4.4724	51.8324	0.5117	56.8166
S5	7.4021	66.3978	0.4966	74.2965	S27	4.3371	51.2370	0.4588	56.0330
S6	6.0958	71.6672	0.7170	78.4800	S28	4.5415	50.5976	0.2339	55.3731
S7	3.7586	48.9386	0.4239	53.1211	S29	4.4737	49.9166	0.6753	55.0656
S8	3.6280	47.3974	0.4379	51.4633	S30	3.0641	39.2108	0.3607	42.6356
S9	6.3182	72.7698	0.8009	79.8889	S31	3.9220	35.4282	0.3748	39.7250
S10	7.5101	77.8618	0.8002	86.1722	S32	3.2070	30.8135	0.4758	34.4962
S11	7.4645	73.4008	0.6682	81.5334	S33	2.3056	30.5546	0.1927	33.0530
S12	7.1861	66.7112	0.8007	74.6980	S34	5.5123	64.3674	0.6635	70.5432
S13	6.9046	66.5628	0.7676	74.2350	S35	4.3085	51.9048	0.6902	56.9035
S14	6.7745	67.4656	0.6808	74.9209	S36	3.3528	33.2218	0.2708	36.8454
S15	6.5587	65.5728	0.4414	72.5729	S37	2.8378	31.8036	0.5329	35.1743
S16	5.9527	63.6874	0.4635	70.1036	S38	4.1026	50.5867	0.4031	55.0925
S17	5.5189	61.8256	0.3310	67.6754	S39	3.6178	35.4944	0.3604	39.4726
S18	5.5354	61.4338	0.3843	67.3535	S40	3.1138	34.0688	0.2205	37.4031
S19	5.4875	68.5241	0.8000	74.8116	S41	3.3494	33.6087	0.3673	37.3255
S20	3.7362	49.2868	0.4576	53.4806	S42	1.5733	30.0889	0.4764	32.1386
S21	3.9933	48.1048	0.4777	52.5759	S43	3.1228	34.9204	0.3976	38.4408
S22	5.9406	60.2024	0.5297	66.6728	S44	3.5734	31.5828	0.2827	35.4389

#### 3.9.1 Principal component analysis (PCA)

Data concerning the contents of the three components were imported into SPSS 27.0 software for PCA. The eigenvalues, variance contribution rates, and comprehensive scores of the PCs were calculated, as shown in Tables 6 and 7. The cumulative variance contribution rate of the first PC was calculated to be 84.599% (greater than 80%), and thus was considered highly representative of the information relating to the three Q-markers in GSB. Therefore, PC1 was selected for quality evaluation. The factor loading matrix values for the swertiamarin, gentiopicroside, and sweroside in PC1 were 0.927, 0.959, and 0.871, respectively. The eigenvector was then derived from the initial factor loading matrix and eigenvalue, after which the PC score *Y* was obtained as *Y* = 0.582*X*_1_ + 0.602*X*_2_ + 0.547*X*_3_, where *X*_1_, *X*_2_, and *X*_3_ are the content standardization results for each of the three components. Finally, taking the variance contribution rate of the first PC as the weight coefficient, the comprehensive score *F* was calculated as *F* = 0.84599*F*_1_.

**TABLE 6:** Eigenvalues and variance contributions.

Ingredients	Initial eigenvalues			Principal factor contribution rate		
	Total	Variance percentage	Variance cumulative	Total	Variance percentage	Variance cumulative
1	2.538	84.599	84.599	2.538	84.599	84.599
2	0.357	11.903	96.502	0.357	11.903	96.502
3	0.105	3.498	100.000	0.105	3.498	100.000

**TABLE 7:** Principal component scores, composite scores and rankings.

NO.	Principal component score	Overall score	Sort	NO.	Principal component score	Overall score	Sort
S1	2.03	1.72	6	S23	2.43	2.06	2
S2	1.51	1.28	13	S24	1.52	1.29	12
S3	0.79	0.67	16	S25	−0.29	−0.25	24
S4	2.05	1.73	5	S26	−0.31	−0.26	25
S5	1.26	1.07	14	S27	−0.53	−0.45	26
S6	1.67	1.41	9	S28	−1.15	−0.98	32
S7	−0.93	−0.79	30	S29	0.1	0.08	21
S8	−1	−0.85	31	S30	−1.75	−1.48	37
S9	2.04	1.72	7	S31	−1.57	−1.32	33
S10	2.66	2.25	1	S32	−1.7	−1.44	35
S11	2.07	1.75	4	S33	−2.87	−2.42	44
S12	2.1	1.77	3	S34	1.01	0.86	15
S13	1.89	1.6	8	S35	0.17	0.14	20
S14	1.63	1.38	10	S36	−2.16	−1.83	41
S15	0.77	0.65	17	S37	−1.62	−1.37	34
S16	0.55	0.46	19	S38	−0.81	−0.68	28
S17	−0.07	−0.06	23	S39	−1.71	−1.45	36
S18	0.08	0.06	22	S40	−2.36	−2	43
S19	1.57	1.33	11	S41	−1.86	−1.57	39
S20	−0.83	−0.7	29	S42	−2.3	−1.95	42
S21	−0.73	−0.61	27	S43	−1.8	−1.52	38
S22	0.6	0.51	18	S44	−2.11	−1.79	40

#### 3.9.2 Entropy-weighting technique for order preference by similarity to ideal solution (TOPSIS)

The entropy method is a mathematical approach for evaluating the degree of dispersion of an index. The greater the dispersion degree, the greater the index’s influence on the comprehensive evaluation score. Thus, entropy values reflect the index’s dispersion, where higher dispersion correlates with a greater impact on the final evaluation. TOPSIS is a multi-attribute decision analysis method that assesses the performance of each scheme by calculating distances to an ideal solution and a negative ideal solution.

##### 3.9.2.1 Normalized decision matrix

For a dataset with multiple evaluation indices (*M*_j_) and *i*th sample (*C*_i_), the performance value is denoted as *X*_ij_ (*i* = 1, 2, 3 … m; *j* = 1, 2, 3 … n). The resulting multi-index decision matrix is represented as *X* = (*x*)_n×m_. Normalized values were obtained using min-max normalization, with the results shown in Table 8.

**TABLE 8:** Normalized raw data of 44 batches of GSB samples.

NO.	Swertiamarin	Gentiopicroside	Sweroside	NO.	Swertiamarin	Gentiopicroside	Sweroside
S1	0.804	0.908	0.847	S23	1.000	0.746	0.987
S2	0.517	0.848	0.999	S24	0.633	0.740	0.969
S3	0.377	0.798	0.835	S25	0.435	0.462	0.519
S4	0.911	0.767	0.867	S26	0.430	0.455	0.525
S5	0.864	0.760	0.500	S27	0.410	0.443	0.438
S6	0.670	0.870	0.862	S28	0.440	0.429	0.068
S7	0.324	0.395	0.380	S29	0.430	0.415	0.793
S8	0.305	0.362	0.403	S30	0.221	0.191	0.276
S9	0.703	0.893	1.000	S31	0.348	0.112	0.299
S10	0.880	1.000	0.999	S32	0.242	0.015	0.465
S11	0.873	0.907	0.782	S33	0.109	0.010	0.000
S12	0.832	0.767	1.000	S34	0.584	0.718	0.774
S13	0.790	0.763	0.945	S35	0.405	0.457	0.818
S14	0.771	0.782	0.803	S36	0.264	0.066	0.128
S15	0.739	0.743	0.409	S37	0.187	0.036	0.559
S16	0.649	0.703	0.445	S38	0.375	0.429	0.346
S17	0.585	0.664	0.227	S39	0.303	0.113	0.276
S18	0.587	0.656	0.315	S40	0.228	0.083	0.046
S19	0.580	0.805	0.999	S41	0.263	0.074	0.287
S20	0.321	0.402	0.435	S42	0.000	0.000	0.466
S21	0.359	0.377	0.469	S43	0.230	0.101	0.337
S22	0.647	0.630	0.554	S44	0.297	0.031	0.148

##### 3.9.2.2 Approximation calculation

After constructing the decision matrix and defining the ideal and negative ideal solutions, the closeness coefficient of each scheme to the ideal solution was calculated using Euclidean distance. The quality of the medicinal materials was then ranked based on this coefficient. The greater the relative proximity degree, the greater the alignment with the ideal solution, and the better the quality of the evaluated medicinal materials, and *vice versa*. The ranking results are shown in Table 9. The Euclidean proximity degrees of the 44 GSB batches of differing origin ranged from 0.043 to 0.953, with S10 exhibiting the highest and S33 the lowest values, consistent with findings from the grey correlation analysis.

**TABLE 9:** Specific gravity calculations for 44 batches of GSB samples.

NO.	Swertiamarin	Gentiopicroside	Sweroside	NO.	Swertiamarin	Gentiopicroside	Sweroside
S1	0.037	0.041	0.034	S23	0.046	0.034	0.040
S2	0.024	0.039	0.041	S24	0.029	0.034	0.039
S3	0.017	0.036	0.034	S25	0.020	0.021	0.021
S4	0.042	0.035	0.035	S26	0.020	0.021	0.021
S5	0.039	0.035	0.020	S27	0.019	0.020	0.018
S6	0.031	0.040	0.035	S28	0.020	0.020	0.003
S7	0.015	0.018	0.015	S29	0.020	0.019	0.032
S8	0.014	0.017	0.016	S30	0.010	0.009	0.011
S9	0.032	0.041	0.041	S31	0.016	0.005	0.012
S10	0.040	0.046	0.041	S32	0.011	0.001	0.019
S11	0.040	0.041	0.032	S33	0.005	0.000	0.000
S12	0.038	0.035	0.041	S34	0.027	0.033	0.031
S13	0.036	0.035	0.038	S35	0.018	0.021	0.033
S14	0.035	0.036	0.033	S36	0.012	0.003	0.005
S15	0.034	0.034	0.017	S37	0.009	0.002	0.023
S16	0.030	0.032	0.018	S38	0.017	0.020	0.014
S17	0.027	0.030	0.009	S39	0.014	0.005	0.011
S18	0.027	0.030	0.013	S40	0.010	0.004	0.002
S19	0.026	0.037	0.041	S41	0.012	0.003	0.012
S20	0.015	0.018	0.018	S42	0.000	0.000	0.019
S21	0.016	0.017	0.019	S43	0.010	0.005	0.014
S22	0.030	0.029	0.023	S44	0.014	0.001	0.006

### 3.10 Hierarchical cluster analysis (HCA)

**FIGURE 7:**
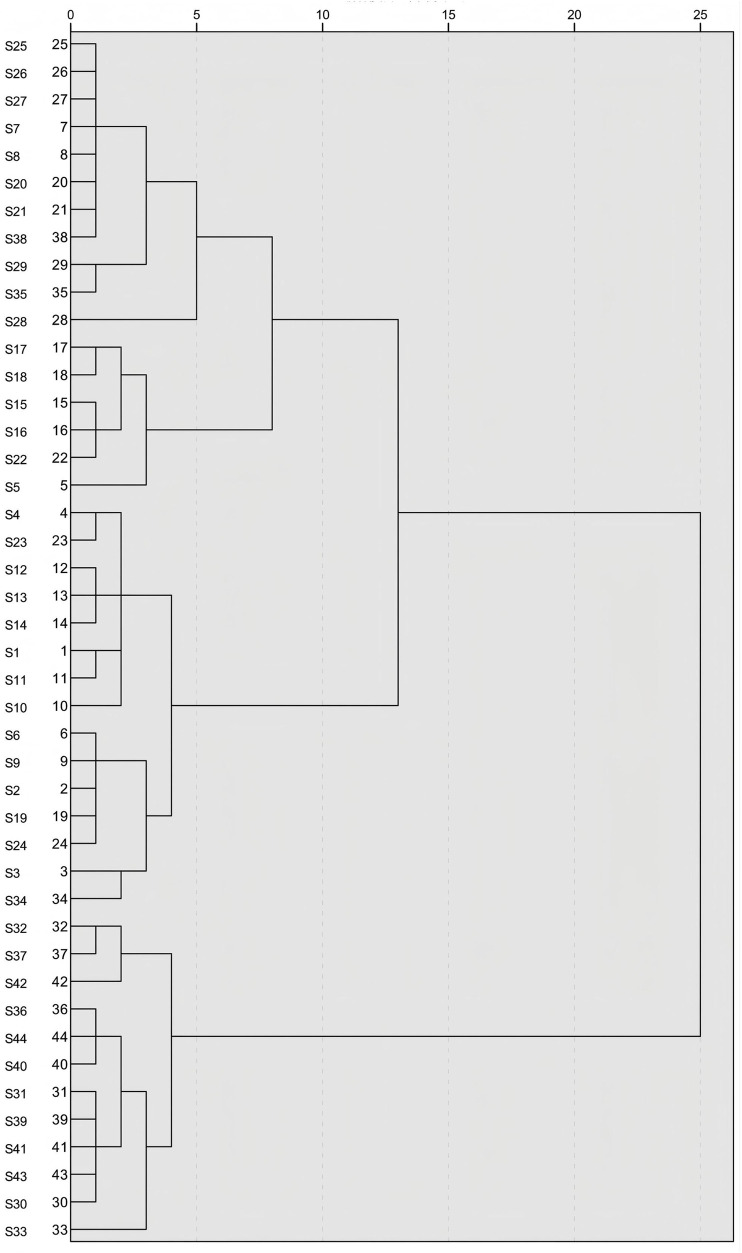
HCA of 44 batches of GSB samples.

After the standardization of the data for the three components and their total content, a systematic clustering analysis with the Euclidean method was performed within SPSS 27.0 software. The dendrogram of clustering results is illustrated in Figure 7. The 44 batches of GSB samples were clustered into three categories. Samples whose comprehensive quality scores, derived from integrating the PC and entropy-weighted TOPSIS methods, ranked highest were almost all in the first category (the top 15 samples). Samples ranked 16–32 based on their comprehensive scores comprised the second category, and the remaining 12 samples constituted the third category. Thus, cluster analysis was effective at categorizing GSB samples by quality. Wild samples from Heilongjiang Province, most wild samples from Liaoning Province, and three batches of cultivated samples were clustered in the first category; wild samples from Inner Mongolia, wild and cultivated samples from Jilin Province, the remaining wild samples, and some cultivated samples from Liaoning Province were grouped in the second category; all remaining cultivated samples from Liaoning Province were grouped in the third category.

## 4 Discussion

In TCM, GSB is characterized as bitter and cold in nature, acting on the liver and gallbladder meridians. Its traditional uses include purging liver and gallbladder fire and promoting sedation. Modern pharmacology supports its broad application in treating diseases of the liver, gallbladder, and heart (Devarbhavi et al., 2023). As the body’s most crucial metabolic organ, the liver is involved in the metabolism of most substances. Abnormal fluctuations in these metabolic substances can damage liver cells, which are the main component of the liver and account for 80% of its mass. Liver diseases cause over 2 million deaths worldwide annually, accounting for 4% of global mortality (Wu S. et al., 2017). Therefore, discovering effective therapeutic agents for liver diseases holds significant clinical importance, and investigating treatments for liver injury is fundamental to managing liver-related disorders.

The concept of Q-markers provides a novel approach to the quality control of TCM. This framework not only reflects the correlation between a TCM’s efficacy and safety but also highlights the unique characteristics of its ingredients (Bahetjan et al., 2023; Li et al., 2021). Compared with traditional methods, an integrated strategy combining fingerprinting and small molecule–protein interaction analysis represents an effective strategy for identifying Q-markers.

To establish a quality standard system for a TCM, its inherent characteristics must be fully considered, necessitating the integration of systems theory and cybernetics for comprehensive evaluation (Zhang et al., 2024). TCM fingerprinting offers a reasonable starting point. Through its ability to describe the overall chemical characteristics of a medicine, this technology is well-suited for establishing modern TCM quality standards (Zhang, 2009).

In this study, HPLC fingerprints were established for 44 batches of GSB sourced from different origins. Twenty-five common peaks were identified, five of which—Peaks 8 (loganic acid), 10 (6′-O-β-D-glucosylgentiopicroside), 11 (swertiamarin), 13 (gentiopicroside), and 15 (sweroside)—were characterized by comparison with standards. Subsequent PCA and OPLS-DA performed on the peak areas confirmed the importance of these compounds. Notably, the VIP values for common peaks 8, 10, 11, 13, 15, and 22 were all greater than 1, establishing these six as the main characteristic components of GSB across producing regions. However, Peak 22 remained unidentified, a limitation that will be addressed in future studies. Consequently, the five identified components—loganic acid, swertiamarin, sweroside, *6*′-*O*-*β*-*D*-glucosylgentiopicroside, and gentiopicroside—were selected as potential Q-markers based on their testability.

Currently, virtual approaches such as network pharmacology and molecular docking are used for screening Q-markers based on effectiveness. However, these methods often rely on public data and lack direct experimental support. Fluorescence spectroscopy offers a critical experimental alternative for studying interactions between proteins and small molecules. Specifically, such interactions can induce alterations in the fluorescence spectra of amino acid residues within the proteins and the small molecules themselves (Li et al., 2024; Shen, 2017). Fluorescence quenching, which is easy to implement and detect, is particularly useful (Zhang, 2016). Changes in fluorescence intensity can reflect the interaction between proteins and small molecules, along with its strength, thereby providing an indication of the potential biological activity of the small molecules (Kaliaperumal et al., 2023). This methodology was successfully applied for the screening of licorice Q-markers through the quantification of the affinity between chemical components and proteins (Liu et al., 2023). In the current study, activity screening experiments showed that different GSB chemical components exhibit varying degrees of interaction with the liver. Based on the principle of ingredient availability, the three components demonstrating the strongest activity—swertiamarin, gentiopicroside, and sweroside—were subsequently screened as potential Q-markers of GSB.

H_2_O_2_ is a strong oxidizing agent capable of inducing lipid peroxidation in the cell membrane along with oxidative damage to DNA, lipids, and intracellular proteins, eventually leading to cell necrosis or apoptosis (Burdon, 1995). Given its advantages, including ease of access and operation, alongside its relative stability, H_2_O_2_ is the most widely used inducer for establishing cell models of oxidative stress and studying cellular oxidative damage (Hurst et al., 2017; Zhu, 2021). Therefore, in this study, we generated an H_2_O_2_-induced NCTC 1469 cell injury model to assess the therapeutic efficacy of the Q-markers of GSB against LI. Compared with normal controls, NCTC 1469 cells treated with H_2_O_2_ exhibited significantly decreased T-AOC and levels of SOD, CAT, and GSH, while the levels of MDA, LDH, ALT, AST, TNF-α, IL-6, and IL-1β displayed the opposite trend. These results indicated that H_2_O_2_ treatment resulted in OS in NCTC 1469 cells, causing significant cell damage and disrupting hepatocyte growth. However, these effects of H_2_O_2_ were greatly mitigated following treatment with swertiamarin, gentiopicroside, or sweroside. Exposure to the Q-markers resulted in concentration-dependent increases in T-AOC and the levels of SOD, CAT, and GSH, accompanied by decreases in the contents of MDA, LDH, ALT, AST, and inflammatory factors (TNF-α, IL-6, IL-1β). Cell damage was also alleviated to a certain extent. These findings demonstrated that the three components are the quality signature components of GSB associated with the treatment of liver injury. However, in this study, Q-marker verification was limited to the cellular level, and although we demonstrated the individual pharmacological activities of swertiamarin, gentiopicroside, and sweroside in the treatment of LI, we did not investigate their potential synergistic effects. This omission creates a discrepancy with the holistic characteristics of TCM, which is fundamentally defined by its multi-component and multi-target effects, and constitutes a major limitation of the study.

To fully align with the core characteristic of TCM, where multiple components exert synergistic effects, subsequent studies will systematically explore the combined actions of these three components. In future research, we will focus on elucidating the key pathways and metabolic nodes regulated by their combination. This will, in turn, help to reveal the comprehensive “component-target-pathway” synergistic action network. This approach will not only address the limitations inherent to single-component studies but also provide a robust scientific basis for investigating the synergistic mechanisms of multi-component TCM and developing clinical compound preparations.

Studies have shown that GSB extract can ameliorate acute LI-induced necrosis, protect the liver, and alleviate alcoholic liver disease in mice *via* the modulation of the TLR4/NF-κB pathway (Cui et al., 2005; Jiang and Xue, 2005; Wang et al., 2023). Gentiopicroside alleviates lipopolysaccharide-induced acute liver failure in mice through its effects on liver-related metabolic enzymes (Lian et al., 2010). Swertiamarin reduces liver toxicity and injury by enhancing the activity of cytochrome P450 3A4 and 2E1 enzymes, along with influencing the protein expression of bile salt export pump (BSEP) and multidrug resistance-related proteins (Wu T. et al., 2017). Sweroside exerts anti-fibrotic effects both *in vivo* and *in vitro* by upregulating the expression of miR-29a and suppressing that of COL1 and TIMP1 (Gong et al., 2020). Furthermore, sweroside improves nonalcoholic fatty liver disease in mice by modulating the expression of the Cd36 and Fgf21 genes, thereby impacting lipid metabolism and the inflammatory response (Yang et al., 2020). Collectively, these findings indicate that swertiamarin, gentiopicroside, and sweroside exert ameliorative effects on liver disease, aligning with our observations.

Our study clearly demonstrated that swertiamarin, gentiopicroside, and sweroside exhibit significant antioxidant and anti-inflammatory activities. Integrating these and previously published findings allows for a more in-depth analysis of the molecular regulatory mechanisms underlying the actions of these compounds. Prior research has demonstrated that the TLR4/MyD88/NF-κB pathway plays a crucial role in hepatic inflammatory responses. Specifically, downregulating the expression of TLR4/MyD88/NF-κB at the protein level reduces the expression of inflammatory factors (TNF-α, IL-6, and IL-1β), thereby mitigating liver injury triggered by inflammation (Yang et al., 2022; Wei M. et al., 2021). Furthermore, swertiamarin exerts a protective effect against carbon tetrachloride-induced liver injury and inflammation in rats through its antioxidant activity, which includes inducing the expression of hepatic detoxifying enzymes and efflux transporters, partially *via* the Nrf2/HO-1 pathway (Wu T. et al., 2017). Separately, sweroside upregulates the expression of SIRT1, consequently inhibiting lipopolysaccharide-induced NF-κB activation and inflammatory response, which effectively lowers the levels of the inflammatory factors TNF-α and IL-1β (Wang et al., 2021). As structurally similar iridoid glycosides, these three compounds are presumably capable of strengthening anti-inflammatory effects by synergistically inhibiting key nodes in the NF-κB pathway. This is consistent with the common characteristic of iridoid glycosides, which typically exert anti-inflammatory effects through the modulation of the NF-κB and MAPK pathways.

The three Q-markers identified in this study—swertiamarin, gentiopicroside, and sweroside—possess clear application value for the quality control, grading, and pharmaceutical development of GSB. At the clinical quality control level, these markers can replace the existing single-component model. A“multi-index threshold standard”can be established based on their combined contents, which avoids deviations in therapeutic efficacy due to fluctuations in a single component. Additionally, in the Chinese medicinal material market, these markers can provide a quantitative basis for establishing graded pricing.

In future drug development, the notable antioxidant and anti-inflammatory activities of these three markers can serve as core quality control indicators for hepatoprotective preparations, thereby guiding the optimization of compound formulation ratios. Concurrently, the correlation between differences in their contents and the ecological conditions of production areas can provide directions for the selection of sites that can serve as bases for the planting of high-quality medicinal material and the optimization of cultivation techniques. This dual approach promotes the full-chain quality standardization of GSB from raw materials to final preparations.

To evaluate the quality of GSB samples from different producing areas, we selected swertiamarin, gentiopicroside, and sweroside, serving as Q-markers for the treatment of liver injury, for content determination and total content calculation among the 44 batches of GSB from the different producing areas. Quantitative analysis revealed significant batch differences in Q-marker content. The concentration ranges for the three components were 1.5733–8.3188 mg/g for swertiamarin, 30.0889–77.8618 mg/g for gentiopicroside, and 0.1927–0.8009 mg/g for sweroside. We established a reliable ranking system using PCA and the entropy-weighted TOPSIS method, and extracted one PC. The best three samples regarding quality were all from wild resources in Liaoning Province, while the three worst were cultivated samples from the same province. HCA showed that the top 15 batches (e.g., S9 and S10), which clustered in the first category, had a total Q-marker content exceeding 60 mg/g. Their gentiopicroside content was 1.5–2 times that of cultivated samples from the same province. This indicated that wild GSB from Heilongjiang and Liaoning had the highest content and best quality across the three Q-marker types. Samples from Inner Mongolia and Jilin exhibited moderate and stable quality; notably, the quality of wild and cultivated samples in Jilin was similar, reflecting the maturity of local cultivation techniques. Conversely, the bottom 12 batches (e.g., S33 and S42) had a total Q-marker content of less than 40 mg/g. Cultivated Liaoning samples were scattered across three clusters, indicating regional quality differences potentially related to variations in cultivation techniques, climate, growth environment, soil pH, and light intensity. Habitat analysis of wild GSB from Liaoning, classified in the second cluster, revealed that it was collected from a shaded, humid understory environment. This suggests that light intensity may affect the accumulation of the three active components in GSB. This ranking system provides a direct basis for quality grading in clinical and industrial applications.

The entropy-weighted TOPSIS method used in this study demonstrated significant advantages in evaluating the quality of GSB. Traditional methods, which rely on single-component quantification or subjective weighting, inherently fail to reflect the synergistic effects of multiple components and are prone to introducing biases. While the TOPSIS approach is suitable for comparative evaluation, its traditional reliance on subjective weights remains a limitation. In this study, we combined the entropy weight method with TOPSIS, achieving objective weighting based on data dispersion and effectively avoiding artificial biases. This is consistent with the component variability observed in multi-batch and multi-origin samples. The integration of this method with PCA and HCA not only objectively weights based on the actual variation of the three quality control components but also generates directly rankable results that distinguish quality, an outcome unattainable with a single method. Furthermore, this approach is innovative compared to studies on other medicinal materials as it correlates GSB quality rankings with liver injury treatment efficacy, establishing a critical link between chemical components and biological function. It provides a reliable framework for multi-index and multi-batch medicinal material quality evaluation, facilitating the standardization of quality assessment.

The above results indicate that wild GSB from Heilongjiang or Liaoning Province should be preferentially selected to ensure high efficacy. Simultaneously, these findings provide clear directions for improving cultivation techniques in Liaoning Province to mitigate quality fluctuations. Notably, while swertiamarin, gentiopicroside, and sweroside are known to possess hepatoprotective properties, in this study, we filled a crucial gap between chemical components and practical quality control by verifying the measurability, regional differentiation, and functional relevance of these compounds. This establishes them as Q-markers with both identification value and efficacy correlation.

In conclusion, in this study, GSB Q-markers (swertiamarin, gentiopicroside, and sweroside) for the treatment of liver injury were predicted by integrating HPLC fingerprinting and “small molecule–protein” interaction models with a multi-component chemical model. Subsequent *in vitro* experiments further verified these predictions, laying an important foundation for follow-up research. However, a major limitation of this work is the lack of *in vivo* verification to further clarify the metabolic behavior and organismal bioavailability of the Q-markers, along with their correlation with overall pharmacodynamics. This limitation needs to be addressed. Based on our *in vitro* research findings, the next step involves systematically undertaking *in vivo* verification by establishing a mouse model of liver injury with oxidative stress. The focus will be on investigating the dynamic change rules of the markers *in vivo*, their targeted action mechanisms, and their correlation with pharmacodynamic indicators. This is expected to comprehensively clarify the scientific connotation of these compounds as Q-markers and provide a more solid experimental basis for the quality control of GSB. Furthermore, the quantification of the Q-markers provided an effective assessment of the differences in quality among GSB samples from different origins, providing a novel approach for identifying GSB quality variations. Given the increasing incidence of liver-related diseases due to the diversification of dietary patterns, exploring the main active components of GSB in liver injury treatment will facilitate the development of ideas and methods for the clinical management of liver injury-related disorders, while promoting GSB quality control research and improving its quality specification system.

## Data Availability

The original contributions presented in the study are included in the article/Supplementary Material, further inquiries can be directed to the corresponding author.

## Glossary

ALT: alanine aminotransferase
AST: aspartate aminotransferase
BCA: Bicinchoninic Acid Assay
CAT: catalase
CCK-8: cell counting kit-8
DMEM: dulbecco’s modified eagle’s medium
DMSO: Dimethyl sulfoxide
D-PBS: dulbecco’s Phosphate-Buffered Saline
GSB: *Gentiana scabra* Bunge.
GSH: glutathione
HCA: hierarchical cluster analysis
**H**_**2**_ **O**_**2**_: Hydrogen peroxide
HPLC: high performance liquid chromatography
HS: horse serum
IL-1β: interleukin-1beta
IL-6: interleukin-6
LDH: lactate dehydrogenase
LC-MS: liquid chromatography-mass spectrometry
LI: liver injury
MDA: malondialdehyde
OPLS-DA: orthogonal partial least square discriminant analysis
OS: oxidativestress
PCA: principal component analysis
PMSF: phenylmethylsulfonyl fluoride
Q-markers: quality markers
RSD: relative standard deviation
SD: standard deviation
SOD: Superoxide Dismutase
T-AOC: total antioxidant capacity
TCM: traditional Chinese medicine
TNF-α: tumor necrosis factor-α
VIP: variable important in projection

## References

[B1] Bahetjan Y. Muhaxi M. Pang K. Kizaibek M. Tang H. Sefidkon F. (2023). Chemistry, bioactivity, and prediction of the quality marker (Q-Marker) of *ferula* plants in China: a review. Mol. (Basel, Switz.) 28 (13), 5191. 10.3390/molecules28135191 37446853 PMC10343754

[B2] Bogdanos D. P. Gao B. Gershwin M. E. (2013). Liver immunology. Compr. Physiol. 3 (2), 567–598. 10.1002/cphy.c120011 23720323 PMC4201126

[B3] Burdon R. H. (1995). Superoxide and hydrogen peroxide in relation to mammalian cell proliferation. Free Radic. Biol. Med. 18 (4), 775–794. 10.1016/0891-5849(94)00198-s 7750801

[B4] Chang B. J. Tang Z. S. Qiu Z. D. (2022). Development of plug-in for quality evaluation and grade prediction of Bupleurum chinense based on “drug efficacy evaluating quality”. Chin. Traditional Herb. Drugs 53 (02), 424–431. 10.7501/j.issn.0253-2670.2022.02.012

[B5] Chen Y. Wen H. Zhou C. Su Q. Lin Y. Xie Y. (2019). TNF-α derived from M2 tumor-associated macrophages promotes epithelial-mesenchymal transition and cancer stemness through the Wnt/β-catenin pathway in SMMC-7721 hepatocellular carcinoma cells. Exp. Cell Res. 378 (1), 41–50. 10.1016/j.yexcr.2019.03.005 30844387

[B6] Cheng J. L. (2017). Pathologic characteristici and screening MicroRNAs biomarkers for liver injury among chronic hepatitis B(CHB)Patients with normal alanine Amiotransferase(PNALT). Zhenjiang: Zhenjiang University.

[B7] Committee National Pharmacopoeia (2020). Pharmacopoeia of People’s Republic of China (Part 1)[S]. Beijing: China Medical Science Press, 99.

[B8] Cui C. X. Liu M. Z. Li T. Z. Zhang X. W. (2005). Study on protecting effects of aqueous extract of the *Gentiana scabra* bye for acute liver injury in rats. J. Med. Sci. Yanbian Univ. (01), 20–22. 10.13422/j.cnki.syfjx.20212103

[B9] Dembek A. Laggai S. Kessler S. M. Czepukojc B. Simon Y. Kiemer A. K. (2017). Hepatic interleukin-6 production is maintained during endotoxin tolerance and facilitates lipid accumulation. Immunobiology 222 (6), 786–796. 10.1016/j.imbio.2017.01.003 28132721

[B10] Devarbhavi H. Asrani S. K. Arab J. P. Nartey Y. A. Pose E. Kamath P. S. (2023). Global burden of liver disease: 2023 update. J. Hepatol. 79 (2), 516–537. 10.1016/j.jhep.2023.03.017 36990226

[B11] Djordjevic A. Spasic S. Jovanovic-Galovic A. Djordjevic R. Grubor-Lajsic G. (2004). Oxidative stress in diabetic pregnancy: SOD, CAT and GSH-Px activity and lipid peroxidation products. J. Maternal-Fetal Neonatal Med. 16 (6), 367–372. 10.1080/14767050400018270 15621558

[B12] Finkel T. Oxidants H. N. J. (2000). Oxidative stress and the biology of ageing. Nature 408 (6809), 239–247. 10.1038/35041687 11089981

[B13] Gong J. Yang F. Yang Q. Tang X. Shu F. Xu L. (2020). Sweroside ameliorated carbon tetrachloride (CCl4)-induced liver fibrosis through FXR-miR-29a signaling pathway. J. Nat. Med. 74 (1), 17–25. 10.1007/s11418-019-01334-3 31280460

[B14] Huai Q. Wang H. (2023). Advance in study of interleukin-1 family cytokines in liver diseases. Chin. Pharmacol. Bull. 39 (05), 828–832. 10.12360/CPB202203021

[B15] Hui Y. J. Yu J. G. Fan X. H. Song Z. X. Tang Z. S. Wang M. (2023). Screening of quality markers and activity verification of Glycyrrhizae Radix et Rhizoma based on small molecule compound-protein interaction. China J. Chin. Materia Medica 48 (20), 5498–5508. 10.19540/j.cnki.cjcmm.20230629.201 38114142

[B16] Hurst J. Kuehn S. Jashari A. Tsai T. Bartz-Schmidt K. U. Schnichels S. (2017). A novel porcine *ex vivo* retina culture model for oxidative stress induced by H_2_O_2_ . Altern. Lab. Animals 45 (1), 11–25. 10.1177/026119291704500105 28409994

[B17] Jang M. K. Kim H. S. Chung Y. H. (2014). Clinical aspects of tumor necrosis factor-α signaling in hepatocellular carcinoma. Curr. Pharm. Des. 20 (17), 2799–2808. 10.2174/13816128113199990587 23944370

[B18] Jiang X. X. Xue B. Y. (2005). Hepatoprotective effects of *Gentiana scabra* on the acute liver injuries in mice. China J. Chin. Materia Medica 30 (14), 1105–1107. 10.3321/j.issn:1001-5302.2005.14.015 16161450

[B19] Kaliaperumal K. Zhang L. Gao L. Xiong Q. Liang Y. Jiang Y. (2023). Insight into the inhibitory mechanisms of hesperidin on α-glucosidase through kinetics, fluorescence quenching, and molecular docking studies. Foods (Basel, Switz.) 12 (22), 4142. 10.3390/foods12224142 38002199 PMC10670601

[B20] Korkmaz A. Ahbab M. A. Kolankaya D. Barlas N. (2010). Influence of vitamin C on bisphenol A, nonylphenol and octylphenol induced oxidative damages in liver of male rats. Food Chem. Toxicol. 48 (10), 2865–2871. 10.1016/j.fct.2010.07.019 20643179

[B22] Li Z. T. Zhang F. X. Fan C. L. Ye M. N. Chen W. W. Yao Z. H. (2021). Discovery of potential Q-marker of traditional Chinese medicine based on plant metabolomics and network pharmacology: periplocae cortex as an example. Phytomedicine 85, 153535. 10.1016/j.phymed.2021.153535 33819766

[B23] Li D. Qu F. Huang Y. Du Y. Yang B. Miao Y. (2024). Spectral analysis of the interaction between ginsenosides Rb3 and bovine serum albumin. Lishizhen Med. Materia Medica Res. 35 (08), 2026–2030. 10.3969/j.issn.1008-0805.2024.08.61

[B24] Lian L. H. Wu Y. L. Wan Y. Li X. Xie W. X. Nan J. X. (2010). Anti-apoptotic activity of gentiopicroside in D-galactosamine/lipopolysaccharide-induced murine fulminant hepatic failure. Chemico-Biol. Interact. 188 (1), 127–133. 10.1016/j.cbi.2010.06.004 20558151

[B25] Liu C. Chen S. Xiao X. Zhang T. Hou W. Liao M. (2016). A new concept on quality marker of Chinese materia medica: quality control for Chinese medicinal products. Chin. Tradit. Herb. Drugs 47 (09), 1443–1457. 10.7501/j.issn.0253-2670.2016.09.001

[B26] Liu C. Liu Y. Tang Z. Song Z. Chang B. Yang X. (2023). Rapid screening of Q-markers for licorice anti-palpitation based on dominance components guidance strategy. Cent. South Pharm. 21 (3), 670. 10.7539/j.issn.1672-2981.2023.03.019

[B27] Malkova A. M. Gubal A. R. Petrova A. L. Voronov E. Apte R. N. Semenov K. N. (2023). Pathogenetic role and clinical significance of interleukin-1β in cancer. Immunology 168 (2), 203–216. 10.1111/imm.13486 35462425

[B28] Newsholme P. Cruzat V. F. Keane K. N. Carlessi R. de Bittencourt P. I. H. Jr (2016). Molecular mechanisms of ROS production and oxidative stress in diabetes. Biochem. J. 473 (24), 4527–4550. 10.1042/BCJ20160503C 27941030

[B29] Peluso I. Morabito G. Urban L. Ioannone F. Serafini M. (2012). Oxidative stress in atherosclerosis development: the central role of LDL and oxidative burst. Drug Targets 12 (4), 351–360. 10.2174/187153012803832602 23061409

[B30] Pinho R. A. Andrades M. E. Oliveira M. R. Pirola A. C. Zago M. S. Silveira P. C. L. (2006). Imbalance in SOD/CAT activities in rat skeletal muscles submitted to treadmill training exercise. Cell Biol. Int. 30 (10), 848–853. 10.1016/j.cellbi.2006.03.011 17011801

[B31] Shen B. J. (2017). Spectral characteristics research of the interaction between the active components of several plants and human serum albumin and DNA. Changchun: Changchun University of Science and Technology.

[B32] Silvestrini A. Meucci E. Ricerca B. M. Mancini A. (2023). Total antioxidant capacity: biochemical aspects and clinical significance. Int. J. Mol. Sci. 24 (13), 10978. 10.3390/ijms241310978 37446156 PMC10341416

[B33] Wang J. Cai X. Ma R. Lei D. Pan X. Wang F. (2021). Anti-inflammatory effects of sweroside on LPS-induced ALI in mice *via* activating SIRT1. Inflammation 44 (5), 1961–1968. 10.1007/s10753-021-01473-4 33913051

[B34] Wang L. Jiang Y. Yu Q. Xiao C. Sun J. Weng L. (2023). Gentiana scabra bge extract (GSE) protects against alcoholic liver disease by regulating the TLR4/NF-κB pathway in mice. Front. Biosci. (Landmark Ed.) 28 (11), 309. 10.31083/j.fbl2811309 38062827

[B35] Wang M. J. Zhang H. L. Chen F. Guo X. J. Liu Q. G. Hou J. (2024). The double-edged effects of IL-6 in liver regeneration, aging, inflammation, and diseases. Exp. Hematol. Oncol. 13 (1), 62. 10.1186/s40164-024-00527-1 38890694 PMC11184755

[B36] Wei M. Li B. Wang Y. Zhao T. He H. Han J. (2021). Protective effect of gentiopicroside against liver injury in mice *via* TLR4/MyD88/NF-κB signaling pathway. Chin. J. Exp. Tradit. Med. Formulae 27 (22), 76–83. 10.13422/j.cnki.syfjx.20212103

[B37] Wei Y. Wang Y. G. Jia Y. Li L. Yoon J. Zhang S. (2021). Liver homeostasis is maintained by midlobular zone 2 hepatocytes. Science 371, eabb1625. 10.1126/science.abb1625 33632817 PMC8496420

[B38] Wu S. Ning Y. Zhao Y. Sun W. Thorimbert S. Dechoux L. (2017). Research progress of natural product gentiopicroside - a secoiridoid compound. Mini Rev. Med. Chem. 17 (1), 62–77. 10.2174/1389557516666160624124127 27342232

[B39] Wu T. Li J. Li Y. Song H. (2017). Antioxidant and hepatoprotective effect of swertiamarin on carbon tetrachloride-induced hepatotoxicity *via* the Nrf2/HO-1 pathway. Cell. Physiology Biochem. 41 (6), 2242–2254. 10.1159/000475639 28448964

[B40] Xu H. L. (2013). Study on the interaction between small-molecular drugs and bovine serum albumin. Changchun: Jilin University.

[B50] Yang Y. Han C. Sheng Y. Wang J. Li W. Zhou X. (2022). Antrodia camphorata polysaccharide improves inflammatory response in liver injury via the ROS/TLR4/NF-κB signal. J. Cell Mol. Med. 26 (9), 2706–2716. 10.1111/jcmm.17283 35352469 PMC9077287

[B41] Yang M. M. Xi X. L. Yang P. (2007). Thermodynamic analysis of the reasonableness of the fluorescence enhancement and quenching theory equations. Acta Chim. Sin. 65 (19), 2109–2116. 10.3321/j.issn:0567-7351.2007.19.005

[B42] Yang Q. Shu F. Gong J. Ding P. Cheng R. Li J. (2020). Sweroside ameliorates NAFLD in high-fat diet induced obese mice through the regulation of lipid metabolism and inflammatory response. J. Ethnopharmacol. 255, 112556. 10.1016/j.jep.2020.112556 31926984

[B43] Zeng Q. Liu Y. Zhan H. Xiao B. Lin M. Chen N. (2021). Quality evaluation of Black ginseng based on HPLC fingerprint and QAMS. Traditional Chin. Drug Res. Clin. Pharmacol. (11), 1710–1715. 10.19378/j.issn.1003-9783.2021.11.016

[B44] Zhang L. J. (2009). Application of chromatographic fingerprint in the quality standard system of traditional Chinese medicine. Anhui Med. Pharm. J. (11), 1414–1417. 10.3969/j.issn.1009-6469.2009.11.055

[B45] Zhang H. (2016). Screening the anticancer drugs against C2H2 domain of human DPF2 using isothermal titration calorimetry and fluorescence quenching. Wuhan: Central China Normal University.

[B46] Zhang K. S. Tian Y. L. (2007). Research and function of catalase in organism. Food Sci. Technol. (01), 8–11. 10.3969/j.issn.1005-9989.2007.01.003

[B47] Zhang H. Zhang Y. Zhang T. Liu C. (2022). Research progress on quality markers of traditional Chinese medicine. J. Pharm. Biomed. Analysis 211, 114588. 10.1016/j.jpba.2022.114588 35091155

[B48] Zhang L. Chen F. Zhang Z. Lin Y. Zhou C. Tang Z. (2024). Prediction of Q-markers in *Perilla frutescens* (L.) Britt based on UPLC fingerprint and network pharmacology. Nat. Prod. Res. Dev. (10), 1800–1812. 10.16333/j.1001-6880.2024.10.016

[B49] Zhu G. Y. (2021). The protective effect and mechanism of short-term low-concentration H2O2 treatment involving ROS-JNK pathway on BRL-3A cells. Guilin: Guilin Medical College.

