# Supplementary material for: Prediction and quality evaluation of quality markers of Gentiana scabra Bunge. in treatment of liver injury
Source: Front Pharmacol. 2025 Nov 6;16:1679981. doi: 10.3389/fphar.2025.1679981 (PMC12630122; doi:10.3389/fphar.2025.1679981)
Supplement: Supplementary file 1 [file Table1.pdf]

Table S1. Peak area of common peaks in the fingerprint

峰号	S1	S2	S3	S4	S5	S6	S7	S8	S9	S10	S11	S12	S13	S14	S15
1	10.070	36.208	55.373	77.019	297.868	107.300	62.541	244.093	128.483	30.675	57.040	207.616	342.370	225.185	33.228
2	12.779	22.937	18.613	21.617	26.493	36.048	28.047	12.743	36.006	16.785	31.878	10.202	23.945	12.366	29.288
3	158.731	165.685	102.680	174.190	155.857	185.211	146.287	136.529	196.493	209.538	176.234	119.567	119.795	98.301	100.677
4	85.791	93.066	138.173	106.261	92.604	226.999	216.778	70.206	227.754	94.263	91.491	108.888	74.340	63.781	59.654
5	314.137	314.618	270.375	546.032	389.203	449.191	272.583	265.751	915.237	451.622	282.322	406.276	315.203	238.429	207.949
6	30.204	34.217	32.942	46.035	47.071	37.508	79.083	59.178	61.805	27.704	61.343	50.885	47.906	30.435	26.530
7	75.970	62.622	31.657	51.790	43.733	53.336	26.962	46.889	21.523	95.034	48.255	46.845	41.116	42.368	33.105
8	7080.582	5498.885	3288.017	2726.696	2907.829	8092.921	3535.251	3481.283	1435.745	6588.808	3812.256	4763.092	2580.203	3564.834	2680.191
9	187.188	187.371	140.756	233.029	197.727	237.498	132.087	137.314	362.341	255.711	164.704	104.952	150.221	99.927	111.925
10	886.323	60.519	777.538	423.525	573.448	503.905	325.341	469.461	983.899	136.651	314.645	688.867	241.019	63.278	90.501
11	1373.687	1376.630	927.586	1501.460	1148.024	1376.745	538.876	598.280	1479.495	1700.884	1355.479	899.485	831.458	816.210	872.668
12	580.998	581.477	396.696	618.488	558.342	529.188	360.314	418.247	617.408	714.492	512.322	454.994	434.082	374.055	371.772
13（S）	19874.521	20447.145	12758.466	18467.303	15632.236	19006.496	16288.793	11504.514	20448.564	23581.238	19152.592	10510.370	11081.686	8707.562	12991.596
14	63.891	46.005	37.347	50.627	82.146	111.587	47.465	43.216	86.818	53.660	43.628	38.353	32.521	32.621	31.358
15	14.401	17.401	44.986	37.758	45.113	51.183	44.332	28.860	112.520	291.206	254.949	29.164	34.668	21.849	245.765

16	85.117	38.063	22.743	48.145	131.632	116.093	36.385	13.827	127.553	31.156	213.816	55.731	152.440	390.838	120.144
17	86.691	13.873	147.068	200.216	143.402	40.803	388.196	57.985	218.430	24.842	104.697	150.670	156.212	122.395	34.865
18	91.830	138.449	103.914	136.568	107.778	97.328	23.292	59.610	205.511	121.280	68.917	84.477	119.653	62.552	80.939
19	125.587	141.776	70.579	58.103	68.600	88.269	95.016	62.923	62.526	136.642	56.212	52.473	66.273	39.272	67.167
20	372.959	329.023	206.507	313.961	267.917	374.694	70.610	137.128	321.572	306.422	261.308	279.549	226.455	225.891	174.883
21	191.406	178.545	160.995	176.898	125.162	103.323	91.079	143.054	34.881	239.648	84.664	81.953	118.274	41.260	72.359
22	1749.623	1551.261	1604.765	2402.891	1740.666	1531.934	28.193	1195.026	1252.290	1999.741	1747.997	1320.192	1560.156	1213.743	1390.271
23	126.399	153.967	94.954	238.039	133.535	89.987	24.959	71.008	153.287	174.517	106.431	83.531	83.233	86.881	78.025
24	77.664	112.417	68.037	124.672	100.372	202.281	145.137	82.953	49.793	122.566	99.566	51.408	60.554	65.637	47.635
25	114.027	221.536	217.259	317.086	229.464	267.820	74.196	274.040	57.911	172.505	183.502	78.694	124.930	90.577	111.873

Table S1. Peak area of common peaks in the fingerprint

峰号	S16	S17	S18	S19	S20	S21	S22	S23	S24	S25	S26	S27	S28	S29	S30
1	17.597	57.536	21.232	27.813	115.879	23.390	26.809	54.236	38.189	13.954	231.008	32.773	151.110	60.117	7.784
2	11.555	26.215	17.345	23.463	33.250	41.725	22.362	20.102	20.462	14.396	12.119	19.344	17.055	26.108	37.908
3	121.413	121.158	125.798	190.593	99.970	105.689	131.013	116.459	138.918	104.882	105.685	121.867	95.759	140.317	116.707
4	72.905	72.530	74.965	104.711	135.141	134.125	74.712	65.292	76.647	54.258	62.106	76.194	98.084	123.579	169.331
5	262.365	310.406	371.422	353.168	396.372	236.685	382.100	320.822	351.287	192.373	231.141	270.170	222.298	311.404	261.477

6	43.391	39.040	37.740	55.324	61.279	47.373	27.047	37.639	37.519	24.847	46.168	20.427	23.244	34.012	44.836
7	34.689	43.348	43.321	86.345	11.114	27.213	50.423	47.477	50.695	45.720	46.202	58.617	29.945	48.154	27.367
8	3084.051	3561.648	2684.537	8491.038	1034.432	3292.306	5415.169	3578.585	3833.738	4127.851	3667.172	3755.629	2017.085	5214.104	2395.306
9	146.998	148.696	184.491	195.990	149.448	131.328	228.451	154.810	173.877	120.619	124.315	149.299	94.597	178.038	128.436
10	86.238	58.028	76.783	32.392	244.073	405.015	107.760	59.113	154.188	61.261	86.642	225.450	617.198	673.047	108.418
11	723.157	1048.394	980.370	1607.509	786.269	747.720	953.853	920.520	1036.885	742.719	819.771	1116.840	579.305	461.345	804.883
12	406.611	545.362	518.142	620.620	247.476	347.923	506.186	489.818	543.884	335.715	388.410	448.993	294.376	663.410	361.429
13（S）	10729.625	15656.804	14938.226	23916.016	7607.469	12495.077	14165.215	13291.758	15528.609	10856.628	10671.035	14518.384	7171.301	14666.764	13987.016
14	25.754	37.635	39.203	87.179	49.255	74.846	32.972	29.951	37.631	26.071	32.072	52.248	24.933	35.399	55.813
15	284.720	263.937	294.052	50.350	40.909	55.720	55.977	298.612	247.431	23.384	24.362	16.478	39.247	53.925	271.299
16	329.431	608.973	73.506	282.977	17.421	52.823	41.200	224.456	245.041	68.732	41.753	18.284	102.963	77.094	41.416
17	44.789	81.983	42.511	43.900	68.818	230.724	22.805	101.882	34.573	20.786	74.713	75.777	87.051	35.810	20.537
18	62.938	78.041	133.729	114.996	44.955	29.253	99.778	68.235	112.216	47.874	59.360	69.551	41.283	71.306	80.276
19	89.241	81.907	104.989	123.841	97.895	103.210	96.919	77.316	106.262	78.255	64.111	86.762	21.877	75.943	80.720
20	248.978	272.120	323.262	402.875	107.481	127.233	259.076	276.038	280.385	198.671	156.752	170.542	106.371	159.616	114.854
21	88.423	128.560	144.559	163.732	34.702	48.444	144.780	140.098	148.259	119.026	110.243	122.883	34.555	120.089	128.899
22	1027.945	1189.382	1444.094	1939.149	724.872	381.928	1282.554	1601.540	1645.986	1091.837	837.561	1119.517	659.303	1122.490	626.863

23	103.983	74.149	105.376	143.696	37.032	76.190	90.750	142.938	94.331	73.868	74.160	82.230	67.803	46.994	35.317
24	68.608	47.689	83.209	83.957	37.626	116.581	91.100	78.044	76.026	71.777	70.447	76.219	42.780	58.961	49.605
25	111.766	86.775	139.300	129.710	53.682	104.569	158.169	168.383	190.503	140.988	165.221	142.526	84.605	119.773	95.936

Table S1. Peak area of common peaks in the fingerprint

峰号	S31	S32	S33	S34	S35	S36	S37	S38	S39	S40	S41	S42	S43	S44
1	6.681	38.385	20.681	38.146	283.671	10.696	7.473	137.724	8.062	9.308	5.578	65.270	9.799	31.647
2	40.475	30.765	25.564	18.987	19.238	23.299	34.608	22.509	21.448	37.571	22.645	32.013	43.495	19.522
3	100.762	90.812	77.448	100.982	142.252	101.332	122.024	180.996	100.145	154.980	83.977	109.318	91.592	91.218
4	220.828	192.992	138.216	61.987	199.703	106.106	167.459	240.093	80.882	270.396	92.404	143.730	138.517	97.520
5	184.950	310.724	194.278	286.308	223.375	229.005	333.939	551.312	262.715	297.523	234.113	347.545	221.227	245.607
6	66.280	46.026	43.593	43.951	29.995	60.430	47.210	54.918	40.156	82.185	56.801	43.194	25.961	43.727
7	26.176	18.874	22.812	28.313	38.051	30.058	30.124	43.337	38.378	37.043	29.929	22.812	25.237	23.858
8	1787.620	2109.113	2310.684	1544.635	2933.676	2754.845	3563.716	2718.979	2768.101	2993.458	3096.221	2559.220	1865.492	1541.205
9	87.304	111.881	97.893	135.072	64.011	149.737	200.022	225.039	118.977	179.185	128.566	167.123	118.870	125.093
10	232.778	306.981	174.405	356.876	571.591	157.977	295.596	637.017	333.767	418.765	156.033	185.305	239.846	175.086
11	511.275	517.217	484.948	788.659	426.268	621.832	673.986	990.459	859.068	545.496	509.851	720.063	657.518	622.055
12	266.552	283.886	235.971	346.375	253.046	298.637	406.202	431.637	346.365	462.269	341.345	357.240	261.836	341.333

13（S）	15744.136	12021.679	8337.498	11312.978	5145.315	9957.640	11863.164	11574.021	12262.649	15821.103	10328.996	9530.366	11440.600	10452.948
14	91.122	35.178	30.018	27.615	29.818	30.086	31.901	100.603	23.293	46.489	20.898	36.681	40.840	32.394
15	262.268	232.569	252.919	264.744	17.490	260.735	237.760	23.802	267.322	257.666	272.148	244.319	291.519	295.539
16	43.891	45.012	49.122	221.584	41.922	62.032	93.357	23.769	97.269	112.628	65.482	104.855	71.206	93.248
17	21.323	28.514	17.104	91.029	182.674	26.965	17.442	59.499	19.093	43.058	18.134	31.433	31.059	18.239
18	54.803	52.823	35.323	62.959	62.165	48.107	68.841	130.095	56.544	34.938	64.567	155.751	63.308	115.280
19	78.688	26.392	83.005	48.874	29.370	116.967	79.769	52.426	45.188	65.116	74.980	93.635	75.574	40.209
20	100.359	92.996	93.582	203.950	188.143	152.403	98.546	257.106	116.310	120.092	94.255	88.757	164.677	159.255
21	116.981	39.252	121.435	92.719	31.195	186.295	137.015	74.099	84.352	212.712	142.255	203.162	68.517	68.573
22	638.249	376.124	380.178	1312.315	1037.690	878.869	762.042	1199.613	945.583	1130.183	634.634	1102.453	679.842	1022.012
23	41.439	31.746	20.809	112.978	105.530	45.146	28.344	93.960	92.267	41.268	24.967	104.788	13.864	61.987
24	53.845	36.567	41.764	59.428	81.253	113.343	45.510	97.238	46.976	20.188	32.989	85.052	23.441	28.632
25	141.379	49.865	56.732	150.184	141.152	242.333	83.151	147.934	114.510	47.362	59.491	260.354	20.294	33.802

Table S2. Retention time of common peaks in the fingerprint

峰号	S1	S2	S3	S4	S5	S6	S7	S8	S9	S10	S11	S12	S13	S14	S15
1	3.373	3.302	3.303	3.306	3.286	3.304	3.344	3.285	3.304	3.303	3.294	3.294	3.283	3.291	3.294

2	5.803	5.780	5.761	5.758	5.774	5.754	5.826	5.778	5.767	5.801	5.770	5.800	5.780	5.780	5.779
3	11.152	11.147	11.113	11.109	11.117	11.104	11.102	11.145	11.116	11.139	11.140	11.154	11.135	11.139	11.135
4	15.145	15.151	15.142	15.116	15.134	15.132	15.124	15.154	15.151	15.132	15.144	15.157	15.144	15.148	15.151
5	16.183	16.172	16.144	16.140	16.153	16.138	16.102	16.185	16.150	16.171	16.182	16.159	16.166	16.173	16.168
6	16.738	16.728	16.699	16.692	16.705	16.681	16.683	16.740	16.700	16.722	16.724	16.725	16.717	16.722	16.723
7	17.556	17.546	17.526	17.516	17.532	17.512	17.468	17.568	17.526	17.547	17.557	17.550	17.543	17.559	17.548
8	19.579	19.585	19.565	19.555	19.564	19.534	19.479	19.597	19.566	19.564	19.591	19.583	19.589	19.607	19.574
9	20.025	20.025	20.006	19.998	20.005	19.984	19.931	20.030	20.008	20.005	20.032	20.014	20.027	20.046	20.013
10	21.022	21.022	21.002	20.990	21.003	20.979	20.904	21.036	20.990	21.006	21.024	21.027	21.035	21.058	21.007
11	21.500	21.500	21.484	21.472	21.481	21.465	21.396	21.514	21.473	21.487	21.506	21.503	21.508	21.531	21.487
12	23.137	23.131	23.112	23.100	23.122	23.098	23.010	23.152	23.119	23.112	23.129	23.135	23.145	23.167	23.119
13（S）	23.716	23.706	23.696	23.676	23.697	23.670	23.596	23.737	23.681	23.682	23.709	23.718	23.726	23.758	23.702
14	24.614	24.601	24.577	24.573	24.594	24.559	24.451	24.627	24.575	24.586	24.608	24.601	24.612	24.635	24.589
15	25.877	25.866	25.849	25.844	25.859	25.829	25.710	25.899	25.849	25.864	25.875	25.881	25.889	25.898	25.860
16	38.748	38.723	38.526	38.713	38.739	38.704	38.662	38.622	38.723	38.521	38.734	38.793	38.798	38.837	38.569
17	42.788	42.743	42.939	42.922	42.956	43.124	42.373	43.009	42.920	42.913	42.925	42.983	43.000	42.999	42.949
18	43.588	43.556	43.574	43.557	43.601	43.566	43.160	43.648	43.557	43.555	43.583	43.630	43.651	43.676	43.586

19	44.371	44.352	44.354	44.344	44.383	44.344	43.985	44.432	44.393	44.347	44.363	44.413	44.429	44.450	44.379
20	45.401	45.375	45.381	45.361	45.417	45.366	45.532	45.452	45.358	45.360	45.388	45.429	45.468	45.483	45.404
21	45.923	45.897	45.899	45.880	45.929	45.878	45.796	45.967	45.880	45.887	45.909	45.944	45.972	45.988	45.924
22	46.260	46.224	46.232	46.209	46.268	46.219	46.693	46.306	46.207	46.213	46.239	46.279	46.317	46.336	46.260
23	48.116	48.085	48.097	48.064	48.132	48.078	47.974	48.165	48.063	48.076	48.099	48.142	48.179	48.201	48.112
24	51.546	51.481	51.503	51.471	51.545	51.477	51.754	51.556	51.463	51.490	51.504	51.551	51.599	51.606	51.526
25	52.227	52.245	52.265	52.235	52.310	52.237	52.369	52.325	52.221	52.246	52.260	52.308	52.363	52.369	52.288

Table S2. Retention time of common peaks in the fingerprint

峰号	S16	S17	S18	S19	S20	S21	S22	S23	S24	S25	S26	S27	S28	S29	S30
1	3.288	3.299	3.296	3.300	3.185	3.302	3.297	3.299	3.300	3.274	3.285	3.287	3.289	3.304	3.234
2	5.766	5.779	5.764	5.764	5.988	5.741	5.763	5.782	5.779	5.694	5.768	5.776	5.796	5.765	6.046
3	11.130	11.143	11.116	11.127	10.318	11.089	11.108	11.126	11.129	10.962	11.097	11.101	11.142	11.120	10.542
4	15.153	15.167	15.129	15.137	13.966	15.123	15.130	15.122	15.143	14.960	15.105	15.116	15.170	15.142	14.232
5	16.167	16.182	16.158	16.170	14.849	16.126	16.145	16.146	16.176	15.995	16.137	16.142	16.178	16.150	15.125
6	16.723	16.738	16.708	16.716	15.437	16.680	16.704	16.707	16.731	16.535	16.702	16.704	16.727	16.708	15.705
7	17.543	17.569	17.536	17.546	16.246	17.510	17.534	17.532	17.556	17.385	17.530	17.536	17.559	17.533	16.468
8	19.593	19.601	19.552	19.562	19.635	19.543	19.576	19.573	19.600	19.442	19.570	19.583	19.603	19.569	18.441

9	20.034	20.039	19.999	20.015	19.635	19.984	20.016	20.011	20.039	19.857	20.006	20.009	20.042	20.010	18.842
10	21.038	21.037	20.997	21.008	20.038	20.979	21.013	21.006	21.042	20.892	21.009	21.009	21.051	21.006	19.839
11	21.510	21.516	21.478	21.487	21.740	21.462	21.492	21.484	21.518	21.341	21.482	21.487	21.525	21.485	20.305
12	23.143	23.154	23.133	23.113	22.293	23.096	23.136	23.128	23.155	23.001	23.124	23.124	23.154	23.118	21.965
13（S）	23.726	23.733	23.712	23.690	23.213	23.672	23.708	23.707	23.733	23.572	23.706	23.705	23.747	23.700	22.533
14	24.615	24.624	24.605	24.604	24.751	24.540	24.595	24.590	24.631	24.479	24.588	24.592	24.633	24.586	23.415
15	25.894	25.896	25.877	25.872	26.159	25.803	25.872	25.862	25.903	25.726	25.859	25.860	25.894	25.859	25.014
16	38.741	38.753	38.721	38.692	37.916	38.965	38.540	38.705	38.742	38.575	38.776	38.562	38.818	38.716	37.906
17	42.798	42.928	43.121	42.926	42.704	42.727	43.096	42.909	42.949	43.153	42.963	42.952	43.001	43.122	42.925
18	43.604	43.601	43.549	43.570	42.937	43.536	43.540	43.564	43.595	43.585	43.607	43.595	43.644	43.559	43.375
19	44.380	44.381	44.338	44.355	43.386	44.308	44.340	44.359	44.380	44.382	44.388	44.386	44.416	44.348	44.067
20	45.403	45.412	45.357	45.380	45.299	45.336	45.348	45.370	45.405	45.410	45.410	45.396	45.440	45.364	45.295
21	45.924	45.933	45.881	45.896	45.687	45.855	45.881	45.891	45.928	45.930	45.931	45.918	45.956	45.888	45.676
22	46.257	46.268	46.209	46.233	46.180	46.190	46.200	46.212	46.259	46.268	46.263	46.253	46.286	46.215	46.178
23	48.122	48.125	48.073	48.096	48.160	48.032	48.057	48.060	48.126	48.128	48.127	48.124	48.137	48.080	48.161
24	51.529	51.551	51.481	51.503	51.742	51.446	51.467	51.464	51.549	51.541	51.535	51.549	51.558	51.471	51.726
25	52.291	52.314	52.243	52.266	52.511	52.200	52.228	52.229	52.313	52.306	52.302	52.309	52.327	52.233	52.508

Table S2. Retention time of common peaks in the fingerprint

峰号	S31	S32	S33	S34	S35	S36	S37	S38	S39	S40	S41	S42	S43	S44
1	3.225	3.228	3.220	3.299	3.296	3.224	3.229	3.294	3.225	3.225	3.220	3.285	3.227	3.223
2	5.928	5.906	5.904	5.803	5.795	5.940	5.946	5.792	5.981	5.929	5.944	5.950	5.928	5.912
3	10.413	10.389	10.386	11.153	11.139	10.431	10.424	11.154	10.391	10.421	10.427	10.428	10.412	10.391
4	14.089	14.072	14.073	15.163	15.180	14.124	14.128	15.190	14.041	14.105	14.123	14.102	14.090	14.073
5	15.000	14.978	14.972	16.176	16.176	15.024	15.020	16.194	14.975	15.009	15.025	15.003	14.995	14.981
6	15.504	15.475	15.475	16.731	16.720	15.530	15.532	16.737	15.485	15.516	15.532	15.504	15.500	15.484
7	16.286	16.260	16.256	17.552	17.544	16.312	16.310	17.572	16.253	16.291	16.310	16.290	16.283	16.274
8	18.239	18.216	18.209	19.591	19.588	18.269	18.271	19.619	18.214	18.252	18.269	18.254	18.243	18.233
9	18.694	18.671	18.667	20.031	20.020	18.724	18.727	20.060	18.664	18.710	18.724	18.712	18.700	18.689
10	19.659	19.638	19.634	21.028	21.040	19.691	19.698	21.059	19.636	19.679	19.687	19.682	19.665	19.654
11	20.138	20.118	20.113	21.507	21.508	20.167	20.175	21.532	20.110	20.160	20.163	20.159	20.145	20.131
12	21.755	21.758	21.746	23.147	23.139	21.791	21.812	23.175	21.740	21.785	21.787	21.801	21.775	21.761
13（S）	22.339	22.328	22.325	23.731	23.732	22.368	22.378	23.756	22.323	22.366	22.368	22.372	22.353	22.342
14	23.223	23.208	23.198	24.616	24.598	23.248	23.256	24.642	23.204	23.252	23.243	23.248	23.232	23.219
15	24.818	24.812	24.800	25.882	25.603	24.839	24.849	25.912	24.797	24.851	24.841	24.848	24.835	24.817
16	37.659	37.646	37.649	38.688	38.821	37.668	37.672	38.630	37.667	37.680	37.669	37.660	37.683	37.661

17	42.678	42.669	42.666	42.935	43.012	42.678	42.685	43.025	42.671	42.695	42.680	42.676	42.696	42.684
18	43.130	43.125	43.121	43.569	43.646	43.136	43.142	43.658	43.123	43.147	43.133	43.128	43.150	43.140
19	43.735	43.738	43.728	44.357	44.416	43.753	43.756	44.437	43.743	43.767	43.749	43.729	43.767	43.752
20	45.036	45.036	45.029	45.381	45.446	45.048	45.055	45.464	45.035	45.058	45.041	45.040	45.059	45.051
21	45.347	45.353	45.346	45.903	45.951	45.368	45.375	45.972	45.355	45.377	45.363	45.354	45.376	45.364
22	45.915	45.918	45.910	46.230	46.302	45.928	45.935	46.316	45.916	45.939	45.922	45.914	45.941	45.929
23	47.880	47.879	47.879	48.083	48.172	47.900	47.897	48.169	47.877	47.914	47.885	47.876	47.904	47.894
24	51.444	51.460	51.450	51.477	51.588	51.454	51.471	51.563	51.452	51.478	51.451	51.445	51.482	51.470
25	52.228	52.233	52.229	52.237	52.356	52.241	52.251	52.326	52.235	52.247	52.232	52.229	52.256	52.247

Table S3. Relative peak area of common peaks in the fingerprint

峰号	S1	S2	S3	S4	S5	S6	S7	S8	S9	S10	S11	S12	S13	S14	S15
1	0.001	0.002	0.004	0.004	0.019	0.006	0.004	0.021	0.006	0.001	0.003	0.020	0.031	0.026	0.003
2	0.001	0.001	0.001	0.001	0.002	0.002	0.002	0.001	0.002	0.001	0.002	0.001	0.002	0.001	0.002
3	0.008	0.008	0.008	0.009	0.010	0.010	0.009	0.012	0.010	0.009	0.009	0.011	0.011	0.011	0.008
4	0.004	0.005	0.011	0.006	0.006	0.012	0.013	0.006	0.011	0.004	0.005	0.010	0.007	0.007	0.005
5	0.016	0.015	0.021	0.030	0.025	0.024	0.017	0.023	0.045	0.019	0.015	0.039	0.028	0.027	0.016
6	0.002	0.002	0.003	0.002	0.003	0.002	0.005	0.005	0.003	0.001	0.003	0.005	0.004	0.003	0.002

7	0.004	0.003	0.002	0.003	0.003	0.003	0.002	0.004	0.001	0.004	0.003	0.004	0.004	0.005	0.003
8	0.356	0.269	0.258	0.148	0.186	0.426	0.217	0.303	0.070	0.279	0.199	0.453	0.233	0.409	0.206
9	0.009	0.009	0.011	0.013	0.013	0.012	0.008	0.012	0.018	0.011	0.009	0.010	0.014	0.011	0.009
10	0.045	0.003	0.061	0.023	0.037	0.027	0.020	0.041	0.048	0.006	0.016	0.066	0.022	0.007	0.007
11	0.069	0.067	0.073	0.081	0.073	0.072	0.033	0.052	0.072	0.072	0.071	0.086	0.075	0.094	0.067
12	0.029	0.028	0.031	0.033	0.036	0.028	0.022	0.036	0.030	0.030	0.027	0.043	0.039	0.043	0.029
13（S）	1.000	1.000	1.000	1.000	1.000	1.000	1.000	1.000	1.000	1.000	1.000	1.000	1.000	1.000	1.000
14	0.003	0.002	0.003	0.003	0.005	0.006	0.003	0.004	0.004	0.002	0.002	0.004	0.003	0.004	0.002
15	0.001	0.001	0.004	0.002	0.003	0.003	0.003	0.003	0.006	0.012	0.013	0.003	0.003	0.003	0.019
16	0.004	0.002	0.002	0.003	0.008	0.006	0.002	0.001	0.006	0.001	0.011	0.005	0.014	0.045	0.009
17	0.004	0.001	0.012	0.011	0.009	0.002	0.024	0.005	0.011	0.001	0.005	0.014	0.014	0.014	0.003
18	0.005	0.007	0.008	0.007	0.007	0.005	0.001	0.005	0.010	0.005	0.004	0.008	0.011	0.007	0.006
19	0.006	0.007	0.006	0.003	0.004	0.005	0.006	0.005	0.003	0.006	0.003	0.005	0.006	0.005	0.005
20	0.019	0.016	0.016	0.017	0.017	0.020	0.004	0.012	0.016	0.013	0.014	0.027	0.020	0.026	0.013
21	0.010	0.009	0.013	0.010	0.008	0.005	0.006	0.012	0.002	0.010	0.004	0.008	0.011	0.005	0.006
22	0.088	0.076	0.126	0.130	0.111	0.081	0.002	0.104	0.061	0.085	0.091	0.126	0.141	0.139	0.107
23	0.006	0.008	0.007	0.013	0.009	0.005	0.002	0.006	0.007	0.007	0.006	0.008	0.008	0.010	0.006

24	0.004	0.005	0.005	0.007	0.006	0.011	0.009	0.007	0.002	0.005	0.005	0.005	0.005	0.008	0.004
25	0.006	0.011	0.017	0.017	0.015	0.014	0.005	0.024	0.003	0.007	0.010	0.007	0.011	0.010	0.009

Table S3. Relative peak area of common peaks in the fingerprint

峰号	S16	S17	S18	S19	S20	S21	S22	S23	S24	S25	S26	S27	S28	S29	S30
1	0.002	0.004	0.001	0.001	0.015	0.002	0.002	0.004	0.002	0.001	0.022	0.002	0.021	0.004	0.001
2	0.001	0.002	0.001	0.001	0.004	0.003	0.002	0.002	0.001	0.001	0.001	0.001	0.002	0.002	0.003
3	0.011	0.008	0.008	0.008	0.013	0.008	0.009	0.009	0.009	0.010	0.010	0.008	0.013	0.010	0.008
4	0.007	0.005	0.005	0.004	0.018	0.011	0.005	0.005	0.005	0.005	0.006	0.005	0.014	0.008	0.012
5	0.024	0.021	0.025	0.015	0.052	0.019	0.027	0.024	0.023	0.018	0.022	0.019	0.031	0.021	0.019
6	0.004	0.003	0.003	0.002	0.008	0.004	0.002	0.003	0.002	0.002	0.004	0.001	0.003	0.002	0.003
7	0.003	0.003	0.003	0.004	0.001	0.002	0.004	0.004	0.003	0.004	0.004	0.004	0.004	0.003	0.002
8	0.287	0.238	0.180	0.355	0.136	0.263	0.382	0.269	0.247	0.380	0.344	0.259	0.281	0.356	0.171
9	0.014	0.010	0.012	0.008	0.020	0.011	0.016	0.012	0.011	0.011	0.012	0.010	0.013	0.012	0.009
10	0.008	0.004	0.005	0.001	0.032	0.032	0.008	0.004	0.010	0.006	0.008	0.016	0.086	0.046	0.008
11	0.067	0.070	0.066	0.067	0.103	0.060	0.067	0.069	0.067	0.068	0.077	0.077	0.081	0.031	0.058
12	0.038	0.037	0.035	0.026	0.033	0.028	0.036	0.037	0.035	0.031	0.036	0.031	0.041	0.045	0.026
13（S）	1.000	1.000	1.000	1.000	1.000	1.000	1.000	1.000	1.000	1.000	1.000	1.000	1.000	1.000	1.000

14	0.002	0.003	0.003	0.004	0.006	0.006	0.002	0.002	0.002	0.002	0.003	0.004	0.003	0.002	0.004
15	0.027	0.018	0.020	0.002	0.005	0.004	0.004	0.022	0.016	0.002	0.002	0.001	0.005	0.004	0.019
16	0.031	0.041	0.005	0.012	0.002	0.004	0.003	0.017	0.016	0.006	0.004	0.001	0.014	0.005	0.003
17	0.004	0.005	0.003	0.002	0.009	0.018	0.002	0.008	0.002	0.002	0.007	0.005	0.012	0.002	0.001
18	0.006	0.005	0.009	0.005	0.006	0.002	0.007	0.005	0.007	0.004	0.006	0.005	0.006	0.005	0.006
19	0.008	0.005	0.007	0.005	0.013	0.008	0.007	0.006	0.007	0.007	0.006	0.006	0.003	0.005	0.006
20	0.023	0.018	0.022	0.017	0.014	0.010	0.018	0.021	0.018	0.018	0.015	0.012	0.015	0.011	0.008
21	0.008	0.009	0.010	0.007	0.005	0.004	0.010	0.011	0.010	0.011	0.010	0.008	0.005	0.008	0.009
22	0.096	0.080	0.097	0.081	0.095	0.031	0.091	0.120	0.106	0.101	0.078	0.077	0.092	0.077	0.045
23	0.010	0.005	0.007	0.006	0.005	0.006	0.006	0.011	0.006	0.007	0.007	0.006	0.009	0.003	0.003
24	0.006	0.003	0.006	0.004	0.005	0.009	0.006	0.006	0.005	0.007	0.007	0.005	0.006	0.004	0.004
25	0.010	0.006	0.009	0.005	0.007	0.008	0.011	0.013	0.012	0.013	0.015	0.010	0.012	0.008	0.007

Table S3. Relative peak area of common peaks in the fingerprint

峰号	S31	S32	S33	S34	S35	S36	S37	S38	S39	S40	S41	S42	S43	S44	RSD%
1	0.000	0.003	0.002	0.003	0.055	0.001	0.001	0.012	0.001	0.001	0.001	0.007	0.001	0.003	144.854
2	0.003	0.003	0.003	0.002	0.004	0.002	0.003	0.002	0.002	0.002	0.002	0.003	0.004	0.002	43.982
3	0.006	0.008	0.009	0.009	0.028	0.010	0.010	0.016	0.008	0.010	0.008	0.011	0.008	0.009	32.220

4	0.014	0.016	0.017	0.005	0.039	0.011	0.014	0.021	0.007	0.017	0.009	0.015	0.012	0.009	63.735
5	0.012	0.026	0.023	0.025	0.043	0.023	0.028	0.048	0.021	0.019	0.023	0.036	0.019	0.023	35.864
6	0.004	0.004	0.005	0.004	0.006	0.006	0.004	0.005	0.003	0.005	0.005	0.005	0.002	0.004	40.621
7	0.002	0.002	0.003	0.003	0.007	0.003	0.003	0.004	0.003	0.002	0.003	0.002	0.002	0.002	35.355
8	0.114	0.175	0.277	0.137	0.570	0.277	0.300	0.235	0.226	0.189	0.300	0.269	0.163	0.147	37.284
9	0.006	0.009	0.012	0.012	0.012	0.015	0.017	0.019	0.010	0.011	0.012	0.018	0.010	0.012	24.815
10	0.015	0.026	0.021	0.032	0.111	0.016	0.025	0.055	0.027	0.026	0.015	0.019	0.021	0.017	88.442
11	0.032	0.043	0.058	0.070	0.083	0.062	0.057	0.086	0.070	0.034	0.049	0.076	0.057	0.060	22.873
12	0.017	0.024	0.028	0.031	0.049	0.030	0.034	0.037	0.028	0.029	0.033	0.037	0.023	0.033	19.439
13（S）	1.000	1.000	1.000	1.000	1.000	1.000	1.000	1.000	1.000	1.000	1.000	1.000	1.000	1.000	0.000
14	0.006	0.003	0.004	0.002	0.006	0.003	0.003	0.009	0.002	0.003	0.002	0.004	0.004	0.003	40.578
15	0.017	0.019	0.030	0.023	0.003	0.026	0.020	0.002	0.022	0.016	0.026	0.026	0.025	0.028	83.733
16	0.003	0.004	0.006	0.020	0.008	0.006	0.008	0.002	0.008	0.007	0.006	0.011	0.006	0.009	105.692
17	0.001	0.002	0.002	0.008	0.036	0.003	0.001	0.005	0.002	0.003	0.002	0.003	0.003	0.002	104.727
18	0.003	0.004	0.004	0.006	0.012	0.005	0.006	0.011	0.005	0.002	0.006	0.016	0.006	0.011	43.983
19	0.005	0.002	0.010	0.004	0.006	0.012	0.007	0.005	0.004	0.004	0.007	0.010	0.007	0.004	36.753
20	0.006	0.008	0.011	0.018	0.037	0.015	0.008	0.022	0.009	0.008	0.009	0.009	0.014	0.015	39.042

21	0.007	0.003	0.015	0.008	0.006	0.019	0.012	0.006	0.007	0.013	0.014	0.021	0.006	0.007	43.380
22	0.041	0.031	0.046	0.116	0.202	0.088	0.064	0.104	0.077	0.071	0.061	0.116	0.059	0.098	38.522
23	0.003	0.003	0.002	0.010	0.021	0.005	0.002	0.008	0.008	0.003	0.002	0.011	0.001	0.006	52.724
24	0.003	0.003	0.005	0.005	0.016	0.011	0.004	0.008	0.004	0.001	0.003	0.009	0.002	0.003	47.361
25	0.009	0.004	0.007	0.013	0.027	0.024	0.007	0.013	0.009	0.003	0.006	0.027	0.002	0.003	56.889

Table S4. Relative retention time of common peaks in the fingerprint

峰号	S1	S2	S3	S4	S5	S6	S7	S8	S9	S10	S11	S12	S13	S14	S15
1	0.146	0.139	0.139	0.140	0.139	0.140	0.145	0.142	0.140	0.139	0.139	0.139	0.138	0.139	0.139
2	0.251	0.244	0.243	0.243	0.244	0.243	0.253	0.250	0.244	0.245	0.243	0.245	0.244	0.243	0.244
3	0.482	0.470	0.469	0.469	0.469	0.469	0.482	0.481	0.469	0.470	0.470	0.470	0.469	0.469	0.470
4	0.655	0.639	0.639	0.638	0.639	0.639	0.657	0.655	0.640	0.639	0.639	0.639	0.638	0.638	0.639
5	0.699	0.682	0.681	0.682	0.682	0.682	0.700	0.699	0.682	0.683	0.683	0.681	0.681	0.681	0.682
6	0.723	0.706	0.705	0.705	0.705	0.705	0.725	0.723	0.705	0.706	0.705	0.705	0.705	0.704	0.706
7	0.759	0.740	0.740	0.740	0.740	0.740	0.759	0.759	0.740	0.741	0.741	0.740	0.739	0.739	0.740
8	0.846	0.826	0.826	0.826	0.826	0.825	0.847	0.846	0.826	0.826	0.826	0.826	0.826	0.825	0.826
9	0.865	0.845	0.844	0.845	0.844	0.844	0.866	0.865	0.845	0.845	0.845	0.844	0.844	0.844	0.844
10	0.909	0.887	0.886	0.887	0.886	0.886	0.908	0.909	0.886	0.887	0.887	0.887	0.887	0.886	0.886

11	0.929	0.907	0.907	0.907	0.906	0.907	0.930	0.929	0.907	0.907	0.907	0.907	0.907	0.906	0.907
12	1.000	0.976	0.975	0.976	0.976	0.976	1.000	1.000	0.976	0.976	0.976	0.975	0.976	0.975	0.975
13（S）	1.025	1.000	1.000	1.000	1.000	1.000	1.025	1.025	1.000	1.000	1.000	1.000	1.000	1.000	1.000
14	1.064	1.038	1.037	1.038	1.038	1.038	1.063	1.064	1.038	1.038	1.038	1.037	1.037	1.037	1.037
15	1.118	1.091	1.091	1.092	1.091	1.091	1.117	1.119	1.092	1.092	1.091	1.091	1.091	1.090	1.091
16	1.675	1.633	1.626	1.635	1.635	1.635	1.680	1.668	1.635	1.627	1.634	1.636	1.635	1.635	1.627
17	1.849	1.803	1.812	1.813	1.813	1.822	1.842	1.858	1.812	1.812	1.810	1.812	1.812	1.810	1.812
18	1.884	1.837	1.839	1.840	1.840	1.841	1.876	1.885	1.839	1.839	1.838	1.840	1.840	1.838	1.839
19	1.918	1.871	1.872	1.873	1.873	1.873	1.912	1.919	1.875	1.873	1.871	1.873	1.873	1.871	1.872
20	1.962	1.914	1.915	1.916	1.917	1.917	1.979	1.963	1.915	1.915	1.914	1.915	1.916	1.914	1.916
21	1.985	1.936	1.937	1.938	1.938	1.938	1.990	1.985	1.937	1.938	1.936	1.937	1.938	1.936	1.938
22	1.999	1.950	1.951	1.952	1.952	1.953	2.029	2.000	1.951	1.951	1.950	1.951	1.952	1.950	1.952
23	2.080	2.028	2.030	2.030	2.031	2.031	2.085	2.080	2.030	2.030	2.029	2.030	2.031	2.029	2.030
24	2.228	2.172	2.173	2.174	2.175	2.175	2.249	2.227	2.173	2.174	2.172	2.173	2.175	2.172	2.174
25	2.257	2.204	2.206	2.206	2.207	2.207	2.276	2.260	2.205	2.206	2.204	2.205	2.207	2.204	2.206

Table S4. Relative retention time of common peaks in the fingerprint

峰号	S16	S17	S18	S19	S20	S21	S22	S23	S24	S25	S26	S27	S28	S29	S30
1	0.139	0.139	0.139	0.139	0.137	0.139	0.139	0.139	0.139	0.139	0.139	0.139	0.139	0.139	0.144
2	0.243	0.244	0.243	0.243	0.258	0.243	0.243	0.244	0.244	0.242	0.243	0.244	0.244	0.243	0.268
3	0.469	0.470	0.469	0.470	0.444	0.468	0.469	0.469	0.469	0.465	0.468	0.468	0.469	0.469	0.468
4	0.639	0.639	0.638	0.639	0.602	0.639	0.638	0.638	0.638	0.635	0.637	0.638	0.639	0.639	0.632
5	0.681	0.682	0.681	0.683	0.640	0.681	0.681	0.681	0.682	0.679	0.681	0.681	0.681	0.681	0.671
6	0.705	0.705	0.705	0.706	0.665	0.705	0.705	0.705	0.705	0.701	0.705	0.705	0.704	0.705	0.697
7	0.739	0.740	0.740	0.741	0.700	0.740	0.740	0.740	0.740	0.738	0.739	0.740	0.739	0.740	0.731
8	0.826	0.826	0.825	0.826	0.846	0.826	0.826	0.826	0.826	0.825	0.826	0.826	0.825	0.826	0.818
9	0.844	0.844	0.843	0.845	0.846	0.844	0.844	0.844	0.844	0.842	0.844	0.844	0.844	0.844	0.836
10	0.887	0.886	0.886	0.887	0.863	0.886	0.886	0.886	0.887	0.886	0.886	0.886	0.886	0.886	0.880
11	0.907	0.907	0.906	0.907	0.937	0.907	0.907	0.906	0.907	0.905	0.906	0.906	0.906	0.907	0.901
12	0.975	0.976	0.976	0.976	0.960	0.976	0.976	0.976	0.976	0.976	0.975	0.975	0.975	0.975	0.975
13（S）	1.000	1.000	1.000	1.000	1.000	1.000	1.000	1.000	1.000	1.000	1.000	1.000	1.000	1.000	1.000
14	1.037	1.038	1.038	1.039	1.066	1.037	1.037	1.037	1.038	1.038	1.037	1.037	1.037	1.037	1.039
15	1.091	1.091	1.091	1.092	1.127	1.090	1.091	1.091	1.091	1.091	1.091	1.091	1.090	1.091	1.110
16	1.633	1.633	1.633	1.633	1.633	1.646	1.626	1.633	1.632	1.636	1.636	1.627	1.635	1.634	1.682

17	1.804	1.809	1.819	1.812	1.840	1.805	1.818	1.810	1.810	1.831	1.812	1.812	1.811	1.819	1.905
18	1.838	1.837	1.837	1.839	1.850	1.839	1.837	1.838	1.837	1.849	1.839	1.839	1.838	1.838	1.925
19	1.871	1.870	1.870	1.872	1.869	1.872	1.870	1.871	1.870	1.883	1.872	1.872	1.870	1.871	1.956
20	1.914	1.913	1.913	1.916	1.951	1.915	1.913	1.914	1.913	1.926	1.916	1.915	1.914	1.914	2.010
21	1.936	1.935	1.935	1.937	1.968	1.937	1.935	1.936	1.935	1.948	1.938	1.937	1.935	1.936	2.027
22	1.950	1.950	1.949	1.952	1.989	1.951	1.949	1.949	1.949	1.963	1.952	1.951	1.949	1.950	2.049
23	2.028	2.028	2.027	2.030	2.075	2.029	2.027	2.027	2.028	2.042	2.030	2.030	2.027	2.029	2.137
24	2.172	2.172	2.171	2.174	2.229	2.173	2.171	2.171	2.172	2.187	2.174	2.175	2.171	2.172	2.296
25	2.204	2.204	2.203	2.206	2.262	2.205	2.203	2.203	2.204	2.219	2.206	2.207	2.204	2.204	2.330

Table S4. Relative retention time of common peaks in the fingerprint

峰号	S31	S32	S33	S34	S35	S36	S37	S38	S39	S40	S41	S42	S43	S44	RSD%
1	0.144	0.145	0.144	0.139	0.139	0.144	0.144	0.139	0.144	0.144	0.144	0.147	0.144	0.144	1.908
2	0.265	0.265	0.264	0.245	0.244	0.266	0.266	0.244	0.268	0.265	0.266	0.266	0.265	0.265	3.917
3	0.466	0.465	0.465	0.470	0.469	0.466	0.466	0.470	0.465	0.466	0.466	0.466	0.466	0.465	1.134
4	0.631	0.630	0.630	0.639	0.640	0.631	0.631	0.639	0.629	0.631	0.631	0.630	0.630	0.630	1.267
5	0.671	0.671	0.671	0.682	0.682	0.672	0.671	0.682	0.671	0.671	0.672	0.671	0.671	0.671	1.359
6	0.694	0.693	0.693	0.705	0.705	0.694	0.694	0.705	0.694	0.694	0.694	0.693	0.693	0.693	1.329

7	0.729	0.728	0.728	0.740	0.739	0.729	0.729	0.740	0.728	0.728	0.729	0.728	0.728	0.728	1.271
8	0.816	0.816	0.816	0.826	0.825	0.817	0.816	0.826	0.816	0.816	0.817	0.816	0.816	0.816	0.954
9	0.837	0.836	0.836	0.844	0.844	0.837	0.837	0.844	0.836	0.837	0.837	0.836	0.837	0.836	0.814
10	0.880	0.880	0.879	0.886	0.887	0.880	0.880	0.886	0.880	0.880	0.880	0.880	0.880	0.880	0.850
11	0.901	0.901	0.901	0.906	0.906	0.902	0.902	0.906	0.901	0.901	0.901	0.901	0.901	0.901	0.878
12	0.974	0.974	0.974	0.975	0.975	0.974	0.975	0.976	0.974	0.974	0.974	0.974	0.974	0.974	0.692
13（S）	1.000	1.000	1.000	1.000	1.000	1.000	1.000	1.000	1.000	1.000	1.000	1.000	1.000	1.000	0.635
14	1.040	1.039	1.039	1.037	1.036	1.039	1.039	1.037	1.039	1.040	1.039	1.039	1.039	1.039	0.726
15	1.111	1.111	1.111	1.091	1.079	1.110	1.110	1.091	1.111	1.111	1.111	1.111	1.111	1.111	1.029
16	1.686	1.686	1.686	1.630	1.636	1.684	1.683	1.626	1.687	1.685	1.684	1.683	1.686	1.686	1.459
17	1.910	1.911	1.911	1.809	1.812	1.908	1.907	1.811	1.912	1.909	1.908	1.908	1.910	1.910	2.311
18	1.931	1.931	1.932	1.836	1.839	1.928	1.928	1.838	1.932	1.929	1.928	1.928	1.930	1.931	2.140
19	1.958	1.959	1.959	1.869	1.872	1.956	1.955	1.871	1.960	1.957	1.956	1.955	1.958	1.958	1.993
20	2.016	2.017	2.017	1.912	1.915	2.014	2.013	1.914	2.017	2.015	2.014	2.013	2.016	2.016	2.266
21	2.030	2.031	2.031	1.934	1.936	2.028	2.028	1.935	2.032	2.029	2.028	2.027	2.030	2.030	2.076%
22	2.055	2.057	2.056	1.948	1.951	2.053	2.053	1.950	2.057	2.054	2.053	2.052	2.055	2.056	2.311%
23	2.143	2.144	2.145	2.026	2.030	2.141	2.140	2.028	2.145	2.142	2.141	2.140	2.143	2.144	2.400%

24	2.303	2.305	2.305	2.169	2.174	2.300	2.300	2.171	2.305	2.302	2.300	2.300	2.303	2.304	2.558%
25	2.338	2.339	2.339	2.201	2.206	2.336	2.335	2.203	2.340	2.336	2.335	2.335	2.338	2.339	2.569%

Table S5. Fluorescence intensity of liver protein interaction with gentiopicrin at different times（ $\bar{x} \pm s$， n =6）

Incubation time（min）	Fluorescence value
0	1990261±10028
10	1365375±11101
20	1320748±20119
30	1328726±10090
40	1254237±21084
50	1334829±10211
60	1330239±20001
70	1204509±11199
80	1310912±10372

Table S6. Different concentrations of drugs interact with liver protein（ $\bar{x} \pm s$， n =6）

Concentration（mg/ml）	gentiopicrin	swertiamarin	sweroside	Loganic acid	6'-*O*-*β*-*D*-Glucosylgentiopicroside
0	5679216±33086	5703550±55663	5764491±55320	5802394±43752	5825349±79385
0.1	5001351±47271	5187619±57508	5289414±61564	5126833±11718	5119315±67444
0.2	4866112±13787	4893613±72441	5131815±34165	5082887±58716	4955755±46821
0.4	4862822±77762	4672404±25826	5124865±232334	5025874±44491	4891552±76332
0.6	4783969±45016	3951665±75675	5065440±21402	5015384±42566	4813413±40515
0.8	4656009±100071	3829670±79328	4901371±58451	4918737±49124	4708078±65260
1	4543971±41586	3789563±45564	4715493±51301	4739606±47581	4101538±59601

Table S7. Percentage Z-score of activity of each drug

Ingredients	Z-score
Gentiopicroside	1.24
Swertiamarin	0.12
Sweroside	0.65
Loganic acid	-0.97
6'-*O*-*β*-*D*-Glucosylgentiopicroside	-1.05

Table S8. The results of peak area in quantitative analysis

NO.	Swertiamarin	Gentiopicroside	Sweroside	NO.	Swertiamarin	Gentiopicroside	Sweroside
S1	814.616	10915.127	141.482	S23	968.299	9767.936	158.698
S2	589.830	10489.096	160.138	S24	680.442	9724.482	156.516
S3	479.869	10134.130	140.063	S25	524.983	7762.661	101.168
S4	898.706	9918.872	143.969	S26	521.091	7717.209	101.860
S5	861.721	9868.584	98.801	S27	505.362	7629.262	91.173
S6	709.832	10646.907	143.355	S28	529.125	7534.830	45.719
S7	438.100	7289.774	84.111	S29	521.244	7434.239	134.912
S8	422.908	7062.140	86.949	S30	357.350	5852.928	71.348
S9	735.690	10809.762	160.307	S31	457.094	5294.225	74.190

S10	874.279	11561.882	160.167	S32	373.961	4612.608	94.592
S11	868.967	10902.962	133.474	S33	269.161	4574.377	37.394
S12	836.604	9914.876	160.256	S34	641.988	9568.691	132.542
S13	803.874	9892.963	153.577	S35	502.035	7727.901	137.931
S14	788.748	10026.312	136.029	S36	390.909	4968.336	53.164
S15	763.656	9746.739	87.643	S37	331.037	4758.857	106.133
S16	693.198	9468.247	92.112	S38	478.091	7533.215	79.915
S17	642.761	9193.252	65.328	S39	421.729	5304.000	71.281
S18	644.683	9135.378	76.107	S40	363.120	5093.442	42.999
S19	639.110	10182.656	160.129	S41	390.522	5025.479	72.677
S20	435.489	7341.218	90.915	S42	184.011	4505.581	94.725
S21	465.385	7166.629	94.993	S43	364.167	5219.227	78.800
S22	691.796	8953.503	105.501	S44	416.560	4726.245	55.584
